# Chitosan and copper nanoparticles in vase solutions elevate the quality and longevity of cut tulips, setting a new standard for sustainability in floriculture

**DOI:** 10.1186/s12870-025-06811-4

**Published:** 2025-06-11

**Authors:** Iman Mohamed El-Sayed, Rasha Ahmed El-Ziat, Eman Zaky Othman

**Affiliations:** 1 Department of Ornamental Plants and Woody Trees, National Research Centre (NRC), Giza, 12622 Egypt; 2 Faculty of Agriculture, Ornamental Horticulture Department, Cairo University, Giza, 12613 Egypt

**Keywords:** *Tulipa gesneriana*, Nanoparticles, Lemongrass, Antioxidant enzyme activity, Microbial growth

## Abstract

Nanoparticles present innovative solutions for postharvest applications, allowing for the development of compounds that effectively extend the vase life of cut flowers by reducing ethylene production and preventing bacterial growth. As a leading choice among cut flowers, Tulips encounter common issues such as neck bending and a limited display life, which can severely impact their marketability. This experiment aims to evaluate the effectiveness of nano chitosan (CHS-NPs) at 3.5 and 7.0 mg L^− 1^, nano copper (Cu-NPs) at 15 and 30 mg L^− 1^, and *Cymbopogon flexuosus* essential oil (LG) at 150 and 300 mg L^− 1^ as innovative, eco-friendly solutions for improving the quality and extending the vase life of cut tulip flowers. The findings reveal that CHS-NPs and Cu-NPs significantly prolong the vase life of cut tulip flowers, with optimal concentrations determined to be 30 mg L^− 1^ Cu-NPs (15.56 and 16.33 days) and 7.5 mg L^− 1^ CHS-NPs (13.01 and 14.00 days), respectively. The greatest RFW% was 110.97 and 112.30% on day 7 in both seasons of cut tulip flowers treated with 30 mg L^− 1^Cu-NPs. These treatments enhance water uptake and relative fresh weight (RFW%), effectively inhibit microbial growth at the stem base, and prevent bacterial blockages in the xylem for up to seven days. Moreover, they substantially increase chlorophyll levels, total soluble carbohydrates, and proteins while decreasing hydrogen peroxide (H_2_O_2_) production, thereby enhancing membrane stability. At the same time, the highest SOD and CAT activity were 1.77 and 1.92 Units mg^− 1^ protein and 2.82 and 2.98 Units mg^− 1^ protein with the 30 mg L^− 1^ Cu-NPs in the first and second seasons, respectively. In conclusion, CHS-NPs at 7.5 mg L^− 1^ and Cu-NPs at 30 mg L^− 1^ significantly enhance the vase life of cut tulips by improving water balance and antioxidant activity, with Cu-NPs demonstrating better effectiveness. Adopting Cu-NPs at the recommended concentration should be prioritized in the tulip floral industry.

## Background

The floriculture industry is very profitable, especially in developing countries. However, it requires careful management and a more environmentally sustainable business model [24, 80]. Tulips (*Tulipa gesneriana* L.) are critical floricultural crops that hold a significant position in the global flower market, ranking third in sales value among cut flowers sold in the Netherlands, following roses and chrysanthemums [70]. With their vibrant colors and delightful fragrances, tulips attract consumers, driving demand and creating trade opportunities. This blooming bulbous plant belongs to the Liliaceae family and boasts over 2,500 cultivars, primarily found in temperate zones [64]. The tulip is also one of the national symbols of the Netherlands, frequently used in landscaping, including flower galaxies and floral arrangements as cut flowers [14]. It is also known as the turban flower due to its large and the multiple layers of vividly colored, warm-toned petals [46]. Tulip cut flowers must be monitored after harvest and subjected to effective procedures such as cooling, pulsing, and storage in floral hydrating preservatives, along with various packing strategies [73]. Hence, caring for all the above increases tulips flower comparatively short vase life, lasting about 7–10 days postharvest by delaying flower senescence, which enhances customer satisfaction, as the longevity of cut flowers significantly impacts their perceived value [65]. Tulips are renowned for their beauty; however, their storage and vase life are limited considerably by early senescence and microbial contamination [70, 76]. These challenges manifest visible wilting symptoms, including bent necks, yellowing leaves, and drooping blooms [36], which collectively reduce their commercial value. Such deterioration is closely linked to postharvest handling practices [68]. Applying floral preservative solutions is considered an effective strategy for extending the vase life of cut flowers, making identifying and optimizing influencing factors a critical focus within the floral industry.

Floral preservatives in holding solutions contribute to cut tulip flower longevity by preserving freshness, promoting water uptake, mitigating oxidative stress, and preventing carbohydrate depletion through antimicrobial properties and energy-supplying effects [67]. Nanoparticles (NPs) are revolutionizing postharvest technology by extending the vase life of fresh-cut flowers through the synthesizing compounds at the nanoscale; these particles offer exceptional properties that reduce the material needed to achieve remarkable results [26, 45]. NPs are non-toxic, cost-effective, and eco-friendly, making them a sustainable choice for floral preservation. Also, their powerful antioxidant and antimicrobial properties further enhance the freshness and longevity of cut flowers [82]. In this context, copper nanoparticles (Cu-NPs) have exhibited antimicrobial and antioxidant properties comparable to those of sodium hypochlorite and nanosilver, making them a promising subject of study [62]. In addition, Cu is a microelement necessary for growth and development and may also have a nourishing effect on vase water [15, 16]. Cu-NPs facilitate easier cell wall interaction and crossing with the intracellular, and principles to promote the development of ROS (reactive oxygen species), thus activating the cell defense mechanism [4, 8]. On the other hand, chitosan (CHS) is a naturally occurring cationic polymer derived from chitin found in insect cuticles, crustacean shells, and fungal cell walls, which is well-known for having broad-spectrum antioxidant and antimicrobial properties [22]. The antimicrobial properties of chitosan are attributed to its positively charged amine groups, which interact with the negatively charged membranes of microbial cells, leading to the leakage of their cellular contents [38]. Additionally, CHS-NPs play a crucial role in extending the vase life of cut flowers by slowing their aging process. They effectively scavenge harmful hydroxyl and superoxide radicals, which protect DNA and enhance antioxidant activities, keeping flowers fresher and more visually appealing for longer [5]. The unique properties of CHS-NPs, such as the quantum size effect, small size, non-toxicity, low cost, and eco-friendly, could make them perform excellent activities [39, 42]. Recently, the option of essential oils (EOs) has been an exciting alternative and novel idea in floral preservatives [74]. These natural organic compounds are complex, volatile mixtures of secondary metabolites, including phenols and their by-products [54]. Because these substances boast impressive antimicrobial properties that effectively prevent the blockage of xylem vessels, they are volatile and safe, making them less hazardous than synthetic alternatives [21, 79]. There are a few discussions on the efficacy of EOs in controlling microbial growth and antioxidant activities for prolonging the vase life of cut flowers [2, 57]. EOs of lemongrass (*Cymbopogon flexuosus*) plants (LG) have high citral, limonene, and geranyl acetate constituents, which are responsible for controlling water pH, microorganism growth, and significant scavenging power against free radicals and their ability to mitigate oxidative damage [61]. Previous studies have demonstrated that CHS-NPs, Cu-NPs, and LG, when applied at various concentrations, can enhance the vase life and quality of cut flowers, such as *Rosa hybrida* treated with 10 mg L⁻¹ of CHS-NPs exhibited enhanced light use efficiency and photosynthetic carbon fixation, leading to extended vase life [63]. In *Gerbera jamesonii*, CHS-NPs (110 nm) improved stem water balance and prevented curvature by suppressing microbial growth [68]. Moreover, CuNPs (20 mg L⁻¹) enhanced fresh weight, reduced bacterial populations, and decreased H₂O₂, increasing vase life by 30% in the cut carnation and chrysanthemum flowers. In another study, Dalda Şekerci et al. [16] reported that 30 ppm Cu-NPs applied to cut Narcissus flowers enhanced fresh weight, water uptake, and vase life, like sodium hypochlorite, due to its antimicrobial properties and nourishing effect on the vase solution. Additionally, cut *Gladiolus grandiflorus* flowers treated with a low concentration of 5 µL L⁻¹ LG as an antimicrobial agent exhibited a notable extension of vase life up to 11 days, along with reduced microbial blockage at the stem end [75]. Therefore, Utilizing CHS-NPs, Cu-NPs, and LG in preservative solutions presents an exciting opportunity to explore their effects on tulip cut flowers, giving attention not only to enhancing the flower quality and antimicrobial activity and inducing the secondary metabolites of tulip cut flower especially it has short vase life and has sales value but also to extend the vase life. Thus, this is the first time to examine the impact of selected nanoparticles (Cu and CHS) and LG, as eco-friendly, sustainable, nontoxic, biodegradable, and biocompatible materials with excellent antimicrobial activity. By focusing on their potential, we aspire to unlock new possibilities for prolonging the longevity and freshness of tulip cut flowers in these preservative solutions.

Our literature review reveals a notable gap in research concerning the use of CHS-NPs, Cu-NPs, and LG as floral preservatives for tulip cut flowers. Despite increasing interest in eco-friendly solutions, there remains a lack of studies focused on sustainable preservatives that can effectively extend the vase life of cut tulips. This study investigates the effectiveness of Cu-NPs, CHS-NPs, and LG at various concentrations to demonstrate how these natural substances can delay senescence while preserving the quality and freshness of the flowers. Ultimately, our goal is to identify the most effective, eco-friendly, and cost-efficient floral preservatives that can be widely applied within the floral industry.

## Methods

### Plant material

Tulip (*Tulipa gesneriana* L.) cv, ‘Apeldoorn Red’ cut flowers used in the experiment were from the commercial growing farm “Floramix Farm” in Kafr Hakim, Giza, Egypt. The flowers were moved to the laboratory of the Ornamental Horticulture Department, Faculty of Agriculture, Giza, Egypt, during two successive seasons, 2022 and 2023, respectively. Flowers were harvested in the same developmental phase with an early opening stage when buds showed full color and were within one day of opening, as Armitage and Laushman [7] recommended. Excess leaves from the lower third of the stem were removed, and the stem was trimmed to approximately 40 cm, retaining three intact leaves along with a basal leaf to facilitate insertion into the glass vessel. Subsequently, tulip stems were recut at 1 cm above the basal plate using a slanted cut under clear distilled water to prevent air embolism and enhance the surface area for improved water uptake. The prepared stems were then placed in 600 mL glass vials containing 500 mL of various vase solutions, according to the treatments. Each holding vase solution contained two cut tulip stems. The glass vessels with stems were placed on laboratory shelves under controlled environmental conditions: a temperature of 20 ± 1 °C, relative humidity of 60 ± 5%, and a 12-hour light/12-hour dark photoperiod. Floral parameters were evaluated daily.

### Floral preservative treatments Preparation

This experiment included examining the influence of one of seven solutions: water with 15 mmol L^− 1^ sucrose (energy source in all treatments) as control (T1), with 3.5 mg L^− 1^ CHS-NPs (T2), 7 mg L^− 1^ CHS-NPs (T3), 15 mg L^− 1^ Cu-NPs (T4), 30 mg L^− 1^ Cu-NPs (T5), 150 mg L^− 1^ LG (T6), or the last one with 300 mg L^− 1^ LG (T7). All solutions were prepared using distilled water and 0.1% (v/v) Tween-20 as a wetting agent and surfactant, sourced from Sigma-Aldrich in St. Louis, MO. These ingredients break down compounds before adding them to the floral preservative solutions. Additionally, surfactants create micro-holes in membranes, facilitating the penetration of macromolecules into cells and ensuring optimal effectiveness [53]. In addition, LG was purchased from the Unity of Squeezing and Extracting Natural Oils at the NRC (National Research Centre) in Doki, Giza, Egypt. Each treatment consisted of three replicates (glass vials), and each glass vial contained two cut tulip stems (2 flowers). The glass vial contains 500 mL of various holding solutions, according to the treatments. The holding solutions were formulated to provide essential hydration and a balanced energy source, thereby enhancing flower longevity by effectively delaying development and senescence. To ensure the reliability of the results and consistency of experimental conditions, all solutions remained unchanged throughout the study. All glass vials containing freshly cut tulip stems in holding solutions were arranged in a completely randomized design, with three replicates and four observational units per treatment in each replicate. The cut tulip stems were monitored daily to evaluate flower longevity and fresh weight. Additionally, the weights of the glass vials containing only water solutions, with and without flowers, were recorded to provide insights into the water relations and hydration status of the flowers. A flower was considered senescent when more than half of the tepal area had wilted, showed discoloration at the edges, and the tepals naturally detached, accompanied by yellowing and wilting of the leaves [35].

### Copper and Chitosan nanoparticles

**Fig. 1:**
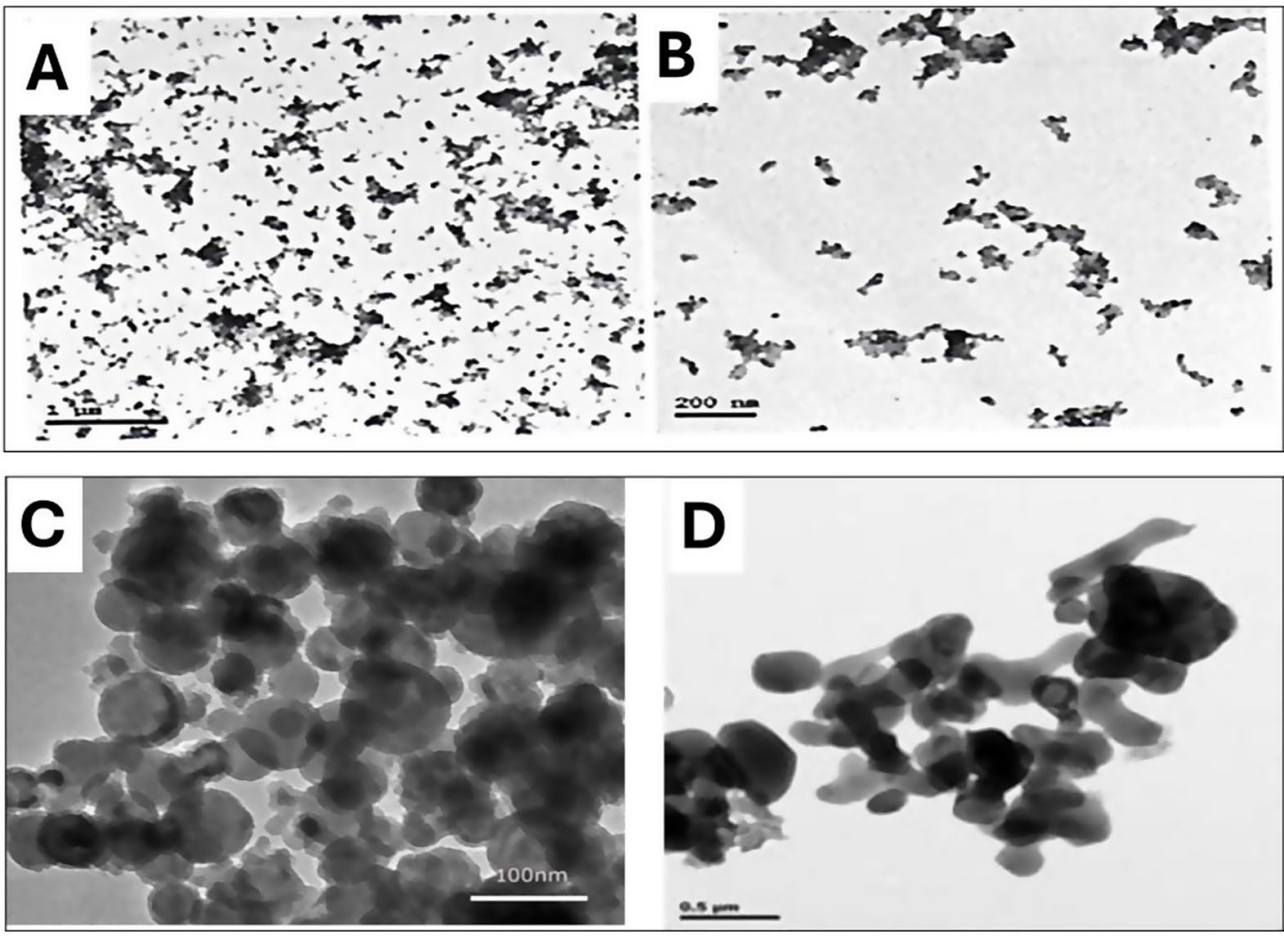
Transmission electron microscopic image of CHS-NPs ranging from 2.0 to 100 nm (**A**, **B**), and Cu-NPs at 100 nm and 0.5 nm (**C**, **D**)

Cu-NPs, sourced from Sigma-Aldrich Chemical Corporation, USA, have demonstrated effectiveness as Zafar et al. [81]. Additionally, we utilized low molecular weight chitosan (over 85% deacetylated), also acquired from Sigma-Aldrich. A chitosan solution was prepared by melting it in a 0.25% acetic acid solution (Merck, Germany). To create CHS-NPs, this was further dissolved into a 1% acetic acid solution, stirred overnight for complete dissolution, and then adjusted with distilled water to the desired volume. The concentration of CHS-NPs was set to 50 nm, following the methods outlined by Khairy et al. [40]. Their properties are summarized in Table 1 and illustrated in Fig. 1.

**Table 1:** Properties of Chitosan and copper as nanoparticles

**Chitosan nanoparticles (CHS-NPs)**	
**CHS-NPs Properties**	
Molecular weight:	Less than 100k Da
Degree of Deacetylation:	85%
Appearance (color):	White
Appearance (form):	Suspension
Avg-size (TEM):	Less than 50 nm
Shape (TEM):	Spherical shape
**Copper nanoparticles (Cu-NPs)**	
**Cu-NPs Properties**	
Batch number:	002
Appearance (color):	Chestnut brown to Black
Avg-size (TEM):	Less than 100 nm
Appearance (form):	Powder
NPs = Nanoparticles	

### Evaluation of traits

#### Vase life

Cut tulip Flowers were meticulously observed daily to evaluate flower longevity and the progression of senescence based on clear indicators of wilting and abscission. This hands-on approach allowed us to accurately estimate vase life by counting the days from the moment the flowers were placed in vase solutions (the first day) until a sizable portion of the tepal and leaf area exhibited severe wilting and the tepals naturally detached (the last day). Additionally, we conducted a thorough visual assessment of flower and leaf coloration, following the methods established by Iwaya-Inoue and Tataka [35]. This comprehensive evaluation ensures that we capture the complete lifecycle and vibrancy of the flowers.

#### Relative fresh weight (RFW%)

The fresh weight of the flowers was determined immediately before immersion in the solutions on the first day. This initial weight was essential for assessing their water status and was measured daily until the end of the vase’s life [37]. The flowers were briefly removed from the solutions for 10 to 20 s to facilitate monitoring without affecting hydration.

$${\text{RFW}}\,{\text{\%=}}\left[{{\text{final}}\,{\text{weight/initial}}\,{\text{weight}}} \right] \times {\text{100}}$$

#### Floral solution uptake

The weights of the glass vials holding only water solutions, without flowers, were recorded every two days during the vase life evaluation [20]. The following formula determines the rate of floral solution uptake:

$${\text{Solution}}\,{\text{Uptake = [(S}}{{\text{t}}_{{\text{ - 1}}}}{\text{ - St)]}}$$

Where St = Solution weight (g) at times 3, 5, and 7 days; St_− 1_ = solution weight (g) on the previous day by El-Sayed and El-Ziat [21].

#### Water loss

To accurately assess water loss, we measured the weight of vases with cut flowers daily during the vase life evaluation period. By the conclusion of this assessment for each treatment, we were able to calculate the total days of water solution loss, providing valuable insights into the hydration of the flowers as:

$${\text{Water}}\,{\text{Loss}}\,\left( {{\text{g/flower/day}}} \right){\text{ = }}\left( {{\text{C}}{{\text{t}}^{{\text{ - 1}}}}{\text{ - Ct}}} \right)$$

Where Ct is the combination of the weight of cut flowers and the vase solution (g) at t = days 2, 4, 6, and Ct^− 1^ is the combination of the weights of cut flowers and vase solution (g) on 0, 2 and 4 days, respectively, by Lü et al. [44].

#### Stomatal conductance and chlorophyll content index (SPAD)

Chlorophyll content was measured in mature leaves seven days after treatment in both seasons. Stomatal conductance was measured in mature leaves on the 3, 5, and 7 days after treatments during both seasons. We used the Minolta Chlorophyll Meter (model SPAD-501) for chlorophyll readings and the LICOR 6400 (Lincoln, Nebraska, USA) for stomatal conductance (µmol H2O m^− 2^s^− 1^), following Khan et al. [41]. Light intensity in the sampling chamber was set to 1500 µmol m^− 2^s^− 1^ using a Li-6400-02B LED light source (LI-COR). Each leaf represented a single replication, with three replications per treatment (*n* = 3).

#### Total soluble carbohydrate and protein

The total level of soluble carbohydrates directly reflects the content of starch and soluble sugars, highlighting its importance. After seven days during the vase-life period, we carefully measured the soluble carbohydrate content of fresh weight (FW) in the petals, adhering to the method of Irigoyen et al. [33]. Furthermore, we determined protein concentration using the reliable Bradford [11] method.

#### Superoxide dismutase and catalase enzyme activities

To effectively assess the health of cut tulip flowers, we recorded the levels of oxidative stress after a seven-day storage period in liquid nitrogen at − 81 °C. Following this critical period, we measured the activity of essential antioxidant enzymes, including superoxide dismutase (SOD) and catalase (CAT), based on the extracted protein content. Therefore, frozen samples were finely ground using mortars placed on an ice bath to maintain their temperature. A total of 100 mg of the resulting powder was then homogenized in an extraction solution composed of 50 mM PBS (plant bio-stimulants), 2% polyvinylpyrrolidone (PVP), 1 mM ethylenediaminetetraacetic acid (EDTA), and 0.05% Triton X-100. Finally, the pH of the solution was carefully adjusted to 7.0 by adding 0.1 N hydrochloric acid (HCl). The supernatant was collected into new tubes following centrifugation at 13,000 rpm for 20 min at 4 °C. We confidently determined SOD activity by measuring the reduction of Nitro Blue Tetrazolium (NBT), as established by Giannopolitis and Ries [27]. We also effectively assessed CAT activity through the decomposition of hydrogen peroxide (H_2_O_2_), following the robust method described by Cakmak and Marschner [12].

#### Quantification of hydrogen peroxide (H_2_O_2_)

After a seven-day vase-life period, the amount of hydrogen peroxide (H_2_O_2_), serving as an oxidative stress marker in fresh petals, was quantified. This assessment was carried out using a Unico UV-2100 spectrophotometer from the USA, which facilitated the measurement of absorption at 390 nm, following the methodology established by Alexieva et al. [3]. This thorough approach ensures an accurate evaluation of the health and quality of the petals.

#### Averages of bacterial counts

In the keeping solution, we incubated the average bacterial infection for 36 h following a seven-day vase life. We diluted samples of preservative solutions (1 mL each) with sterilized distilled water and placed them in Petri dishes. Each dish received 10 mL of sterilized plate agar medium and peptone water, which was stirred briefly for 5 to 10 s and incubated for 2 days at 30 °C. Finally, we counted the bacterial colonies based on CFU/mL, following the method of Marousky [47].

#### Scanning electron microscopy (SEM)

Upon completing the experiment, we conducted a detailed microscopic analysis to investigate the xylem obstruction caused by bacteria at the base of the stems of cut tulip flowers. As Bozzola and Russell [10] highlighted, such analysis is a powerful tool for visualizing floral structures and associated microorganisms. Following Spricigo et al. [69], 2 mm basal tulip stem segments were excised using sharpened stainless-steel blades sterilized with 70% ethanol. Samples were fixed for 1 h in a 2% paraformaldehyde and 2.5% glutaraldehyde solution, then in 0.05 M cacodylate buffer with 0.001 M CaCl₂ (pH 7.2), using a fixative volume ten times that of the sample volume. After that, residual cell gases were removed, and samples were refrigerated until drying. Then, they were washed with 0.05 M cacodylate buffer (pH 7.2) and dehydrated in graded acetone (30%, 50%, 70%, 90% for 10 min each; 100% acetone three times for 10 min). After drying, the samples were stored in a desiccator until examination using a JEOL JEM-1400 transmission electron microscope (TEM).

### Statistical analysis

The experiment followed a completely randomized design with seven treatments, including a control and 6 treatments. Each treatment consisted of three replicates (glass vials), and each glass vial contained two cut tulip stems (2 flowers). Statistical analysis was conducted using analysis of one-way variance (ANOVA) at a 5% significance level in SPSS software, version 21 (Armonk, NY, USA). To determine significant differences among treatments, Duncan’s multiple range test was applied at *p* ≤ 0.05, following the method described by Duncan [18]. All results are presented as the means of three replicates. Additionally, correlations between the seven treatments and traits associated with the antioxidant defense system and carbohydrate and protein content in cut tulip flowers were visualized using heatmaps generated via the ClustVis online tool [49].

## Results

### Vase life

**Fig. 2:**
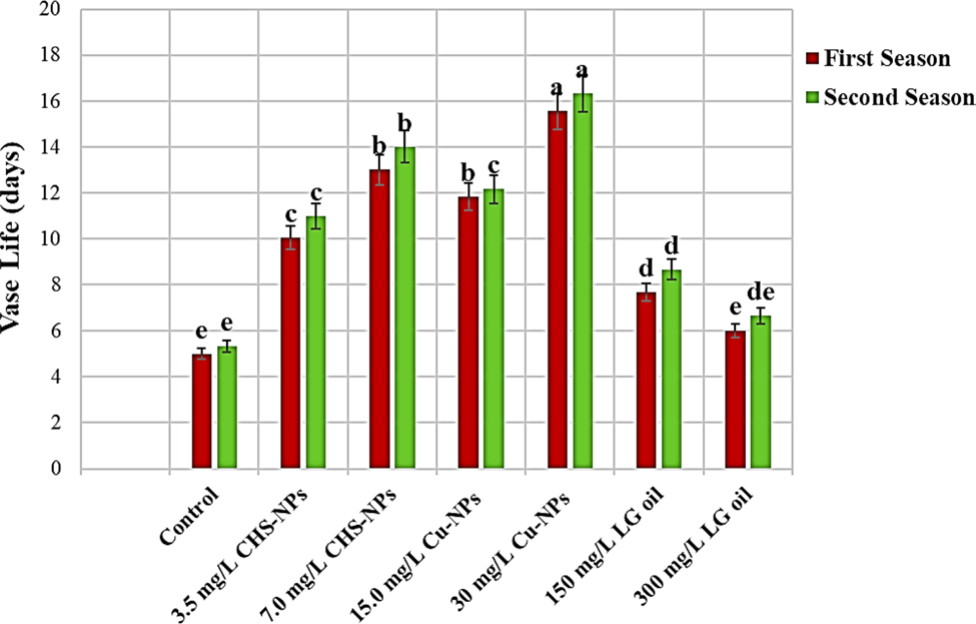
Illustrates the significant impact of varying concentrations of CHS-NPs, Cu-NPs, and LG on enhancing the vase life of cut tulips over two seasons. Error bars represent the SE of the mean, analyzed using Duncan’s multiple range test at a significance level of *p* ≤ 0.05 (ANOVA and Duncan’s multiple range test)

The vase life of tulip cut flowers in various applications of floral solutions showed significantly (*p* = 0.0001) in Fig. 2. Among the seven solutions analyzed, the standout performer was 30 mg L^− 1^ Cu-NPs, which achieved an impressive vase life by (176.68% and 157.9%) compared with the control in the first and second seasons, respectively. Following was 7 mg L^− 1^ CHS-NPs, with a vase life by (129.26% and 121.1%) comparable to untreated cut flowers in the first and second seasons, respectively. Notably, the lower concentration of LG proved to be more effective in prolonging vase life than its higher concentration in both seasons. These highlight the importance of selecting a well-preserved solution to maximize the longevity of cut tulip flowers. In contrast, the shortest vase life for cut flowers was observed in the control group and when using LG at a concentration of 300 mg L^− 1^ during the first and second seasons, respectively. The vase life of a flower is a critical factor that indicates its overall quality, often influencing consumer choices in the floral market. This study revealed that tulip cut flowers treated with Cu-NPs showed the most extended vase life, highlighting the potential of this treatment to enhance floral longevity.

### Relative fresh weight

**Fig. 3:**
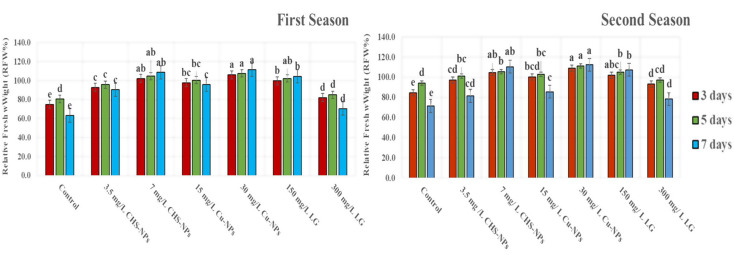
Illustrates the significant impact of varying concentrations of CHS-NPs, Cu-NPs, and LG on enhancing the Relative fresh weight (RFW%) of cut tulips over two seasons. Error bars represent the SE of the mean, analyzed using Duncan’s multiple range test at a significance level of *p* ≤ 0.05 (ANOVA and Duncan’s multiple range test)

The impact of various concentrations of CHs-NPs, Cu-NPs, and LG on the relative fresh weight (RFW%) of tulip cut flowers is truly remarkable throughout their vase life. Our findings reveal that the RFW% of tulip flowers consistently increased until the fifth day across all treatments. Notably, on day 7, while RFW% declined for most treatments, the Cu-NPs at 30 mg L^− 1^ emerged as the most effective, followed closely by CHs-NPs at 7 mg L^− 1^, then LG at 150 mg L^− 1^ (Fig. 3). This underscores the potential of these treatments to enhance the freshness of cut flowers. RFW% achieved an impressive 110.97 and 112.30% with 30 mg L^− 1^ of Cu-NPs on day 7 of the first and second seasons, respectively. In comparison, control flowers treated with distilled water only reached 69.78% and 72.47% in the first and second seasons, respectively. Also, the lowest RFW% was observed in control on all days. These results are like the vase life parameters mentioned above. This significant difference underscores the effectiveness of Cu-NPs in promoting flower quality.

### Water relationship

#### Floral water uptake

**Fig. 4:**
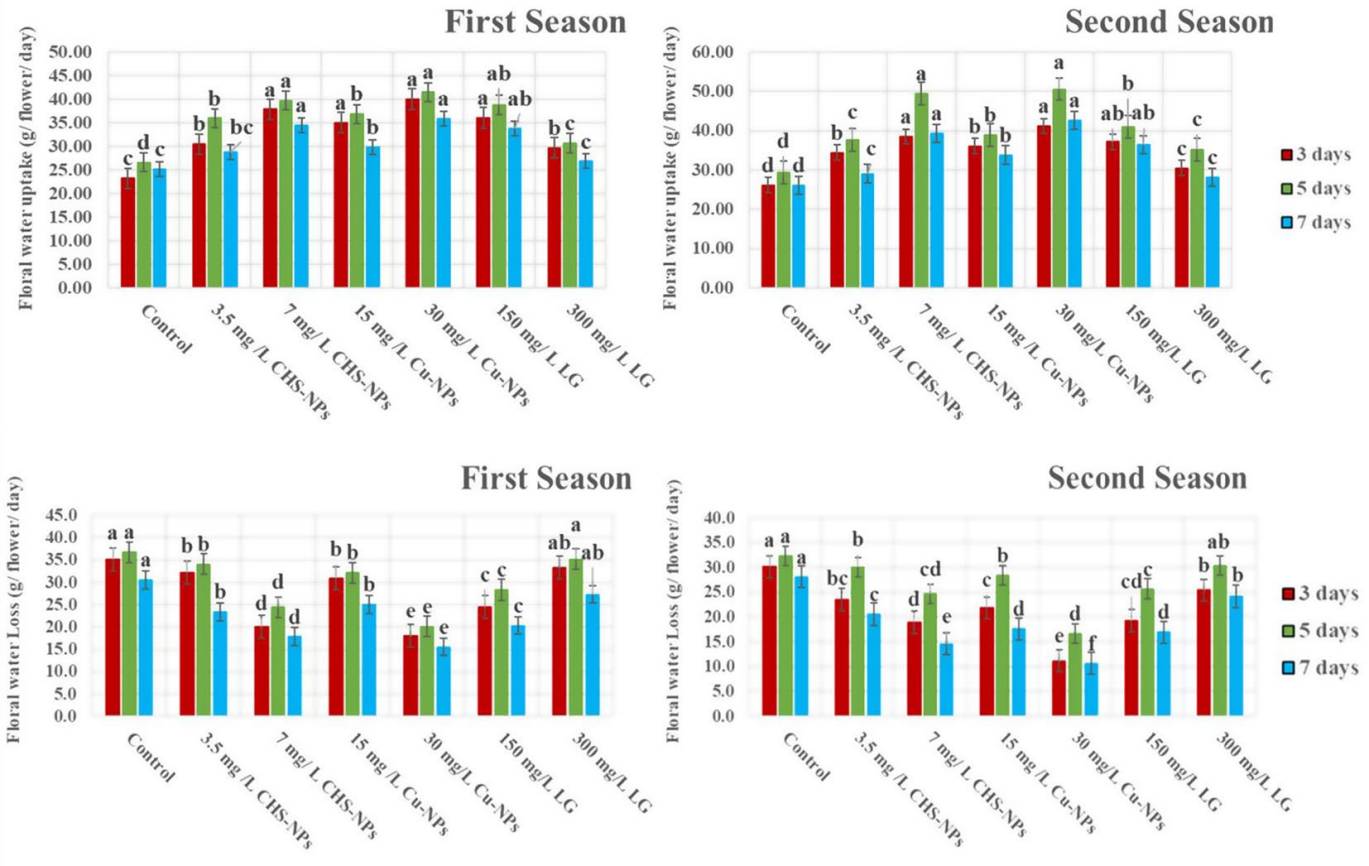
Illustrates the significant effects of varying the concentrations of CHS-NPs, Cu-NPs, and LG on water uptake and water loss in cut tulips over two seasons. Error bars represent the SE of the mean, analyzed using Duncan’s multiple range test at a significance level of *p* ≤ 0.05 (ANOVA and Duncan’s multiple range test)

Floral water uptake exhibited a notable improvement over the vase life period. As shown in Fig. 4 treatment with 30 mg L⁻¹ of Cu-NPs resulted in the most significant increase in water uptake on days 3, 5, and 7 in both seasons. In contrast, the control group (distilled water) consistently demonstrated the lowest water uptake on all measured days. Water uptake increased significantly (*p* = 0.0003) until day 5 across most treatments, followed by a decline, except for CHS-NPs at 7 mg L⁻¹, Cu-NPs at 30 mg L⁻¹, and LG oil at 150 mg L⁻¹. These findings highlight the superior effectiveness of these treatments in promoting water uptake, thereby maintaining flower freshness and extending vase life.

#### Water loss

**Fig. 5:**
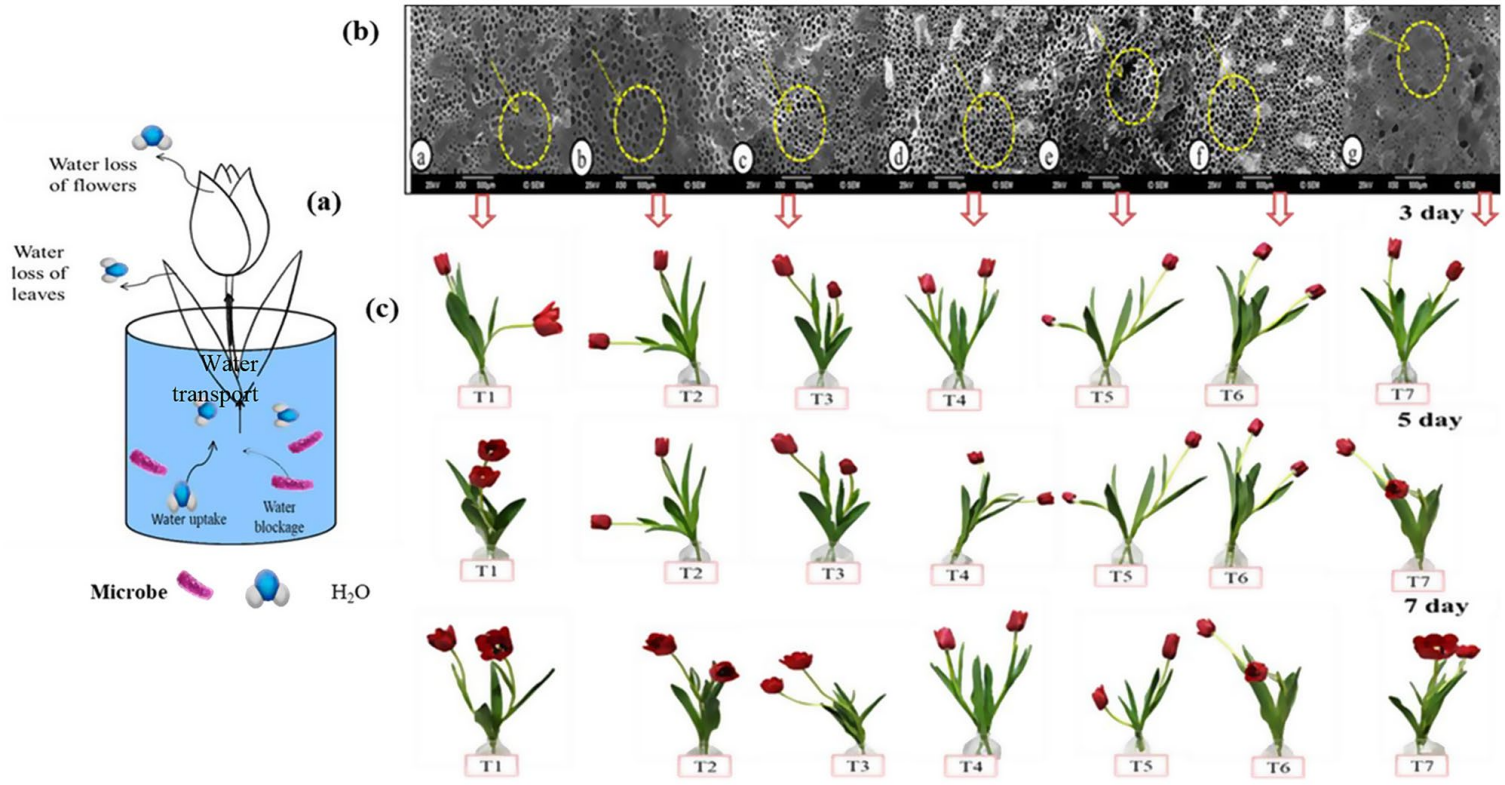
(**a**) Schematic of water relationship in tulip cut flowers, (**b**) Stem ends of tulip cut flowers in different concentrations of Cu-NPS, CHS-NPs, and LG at 7 days, (**c**) Impact of optimal level of Cu-NPs, CHS-NPs, and LG on the vase life of cut tulip during 3,5, and 7 days. T1: control, T2: 7 mg L^− 1^ CHS-NPs, T3: 15 mg L^− 1^ CHS-NPs, T4: 15 mg L^− 1^Cu-NPs, T5: 30 mg L^− 1^Cu- NPs, T6:150 mg L^− 1^ LG, T7: 300 mg L^− 1^ LG

The water loss levels of tulip cut flowers (Figs. 4 and 5) across 3, 5, and 7 days demonstrate a significant reduction with all treatments applied, particularly with CHS-NPs at 7 mg L^− 1^, Cu-NPs at 30 mg L^− 1^, and LG at 150 mg L^− 1^ when compared to untreated flowers. The control treatment exhibited the highest water loss, recording 35.10, 36.75, and 30.59 g/flower/day on days 3, 5, and 7 in the first season, respectively. In the second season, these figures were 30.12, 32.29, and 28.13 g/flower/day. This data highlights the effectiveness of the treatments in promoting better water retention in tulip cut flowers.

#### The correlation between water content, microbial activity, and vase life

Fig. 5 illustrates the notable correlation between water content in cut tulips and the cleanliness of xylem vessels at the stem ends, an association that significantly influences floral longevity. Water uptake generally increased until days 3 to 5 across most treatments, followed by a decline. However, treatments with 7 mg L⁻¹ CHS-NPs, 30 mg L⁻¹ Cu-NPs, and 150 mg L⁻¹ LG maintained elevated water uptake through day 7, indicating sustained hydration. In contrast, the control treatment exhibited the highest rate of water loss on days 3, 5, and 7 (a). These findings highlight the critical role of xylem integrity and microbial control in maintaining water balance and extending vase life. This relationship was further supported by microscopic observations, which revealed significant bacterial accumulation in the xylem vessels of control flowers. Conversely, flowers treated with CHS-NPs (7 mg L⁻¹), Cu-NPs (30 mg L⁻¹), and LG (150 mg L⁻¹) exhibited cleaner xylem vessels with markedly reduced bacterial colonization (b). As a result, these treatments contributed to the prolonged vase life of the cut tulips, while those in the control group showed significantly reduced longevity (c).

**Fig. 6:**
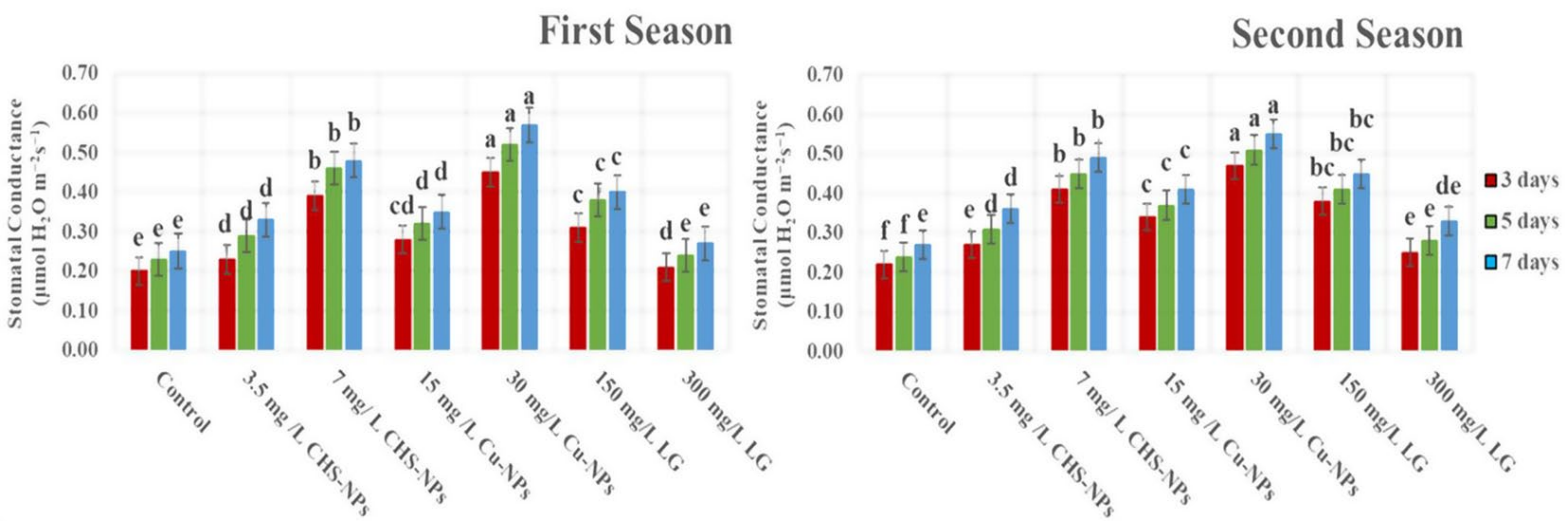
Illustrates the significant impact of varying concentrations of CHS-NPs, Cu-NPs, and LG on enhancing the stomatal conductance of cut tulips over two seasons. Error bars represent the SE of the mean, analyzed using Duncan’s multiple range test at a significance level of *p* ≤ 0.05 (ANOVA and Duncan’s multiple range test)

### Stomatal conductance

The data presented in Fig. 6 reveals significant variation in stomatal conductance throughout the experiment across both seasons. Compared to all other treatments, treatments with 30 mg L⁻¹ Cu-NPs and 7 mg L⁻¹ CHS-NPs resulted in the highest stomatal conductance in cut tulip leaves on days 3, 5, and 7. This finding highlights the critical role stomatal conductance plays in regulating gas diffusion. The enhanced gas exchange between leaf tissues and the atmosphere contributed to significant improvements in photosynthetic attributes, the potential of these treatments to optimize flower quality, and their postharvest performance.

### Total chlorophyll index (SPAD)

**Fig. 7:**
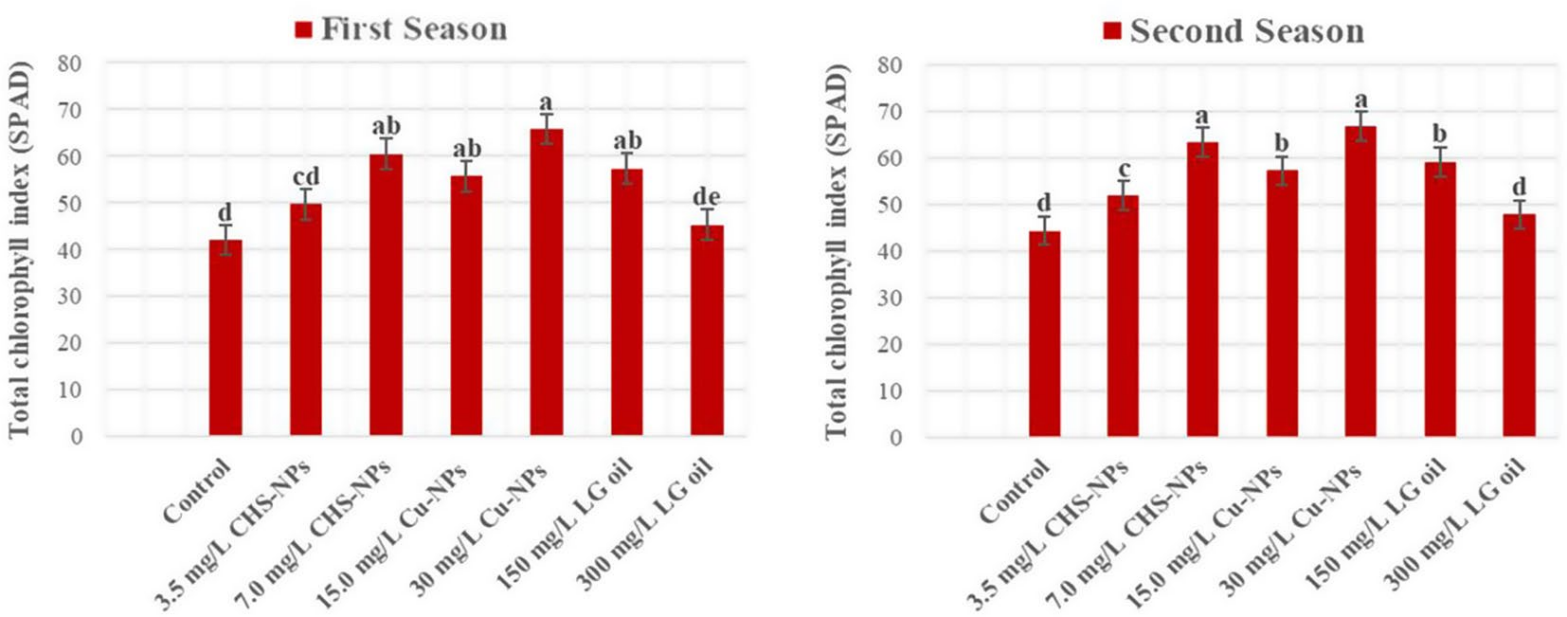
Illustrates the significant impact of varying CHS-NPs, Cu-NPs, and LG on enhancing the total chlorophyll index (SPAD) in cut tulips over two seasons. Error bars represent the SE of the mean, analyzed using Duncan’s multiple range test at a significance level of *p* ≤ *0.05* (ANOVA and Duncan’s multiple range test)

SPAD measurements revealed significantly (*p* = 0.0001) differences among the treatments applied to cut tulip leaves (Fig. 7). As indicated by SPAD values, total chlorophyll content was significantly higher in the Cu-NPs treatment at 30 mg L⁻¹, followed by the CHS-NPs treatment at 7 mg L⁻¹, which outperformed the control in both experimental seasons. In contrast, the control (distilled water) and the LG treatment at 300 mg L⁻¹ exhibited markedly lower total chlorophyll content compared to the other treatments. This highlights that enhancing chlorophyll concentration is not just beneficial but essential for improving the CO_2_ assimilation rate, ultimately contributing to healthier and more vibrant cut tulip leaves.

### Total soluble carbohydrates

**Fig. 8:**
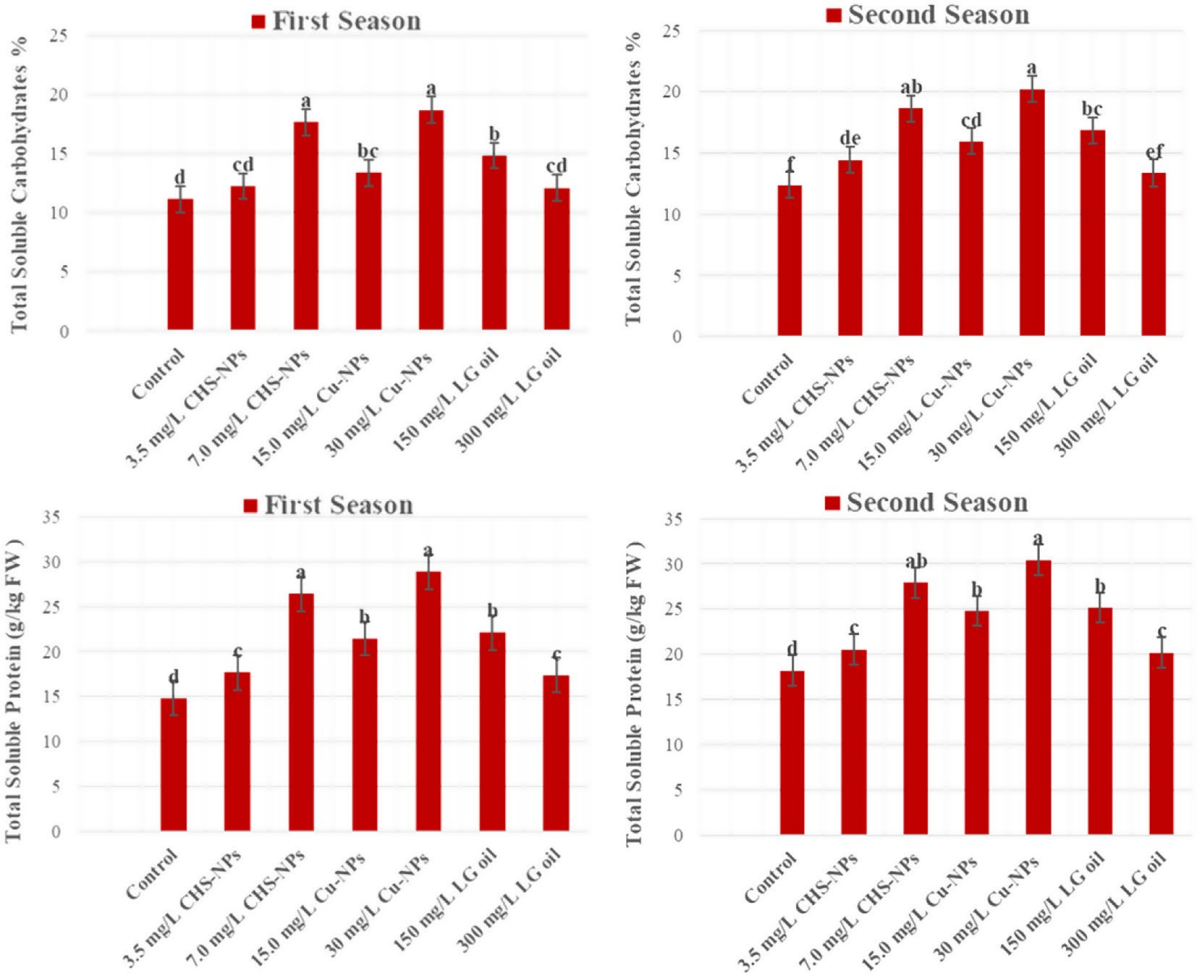
Illustrates the significant impact of varying concentrations of CHS-NPs, Cu-NPs, and LG on the total soluble carbohydrates and protein content in cut tulips over two seasons. Error bars represent the SE of the mean, analyzed using Duncan’s multiple range test at a significance level of *p* ≤ 0.05 (ANOVA and Duncan’s multiple range test)

The total soluble carbohydrates measured in flowers showed significantly (*p* = 0.0001) with Cu-NPs, CHS-NPs, and LG oil are shown in Fig. 8. Remarkably, the flowers exposed to Cu-NPs at a concentration of 30 mg L^− 1^ achieved impressive carbohydrate levels of 18.66% and 20.21% across both seasons, outshining all other treatments. Similarly, those treated with CHS-NPs at 7 mg L^− 1^ demonstrated significantly increased carbohydrate levels of 17.70% and 18.65% in both seasons, further showcasing the remarkable effectiveness of these NPs in boosting carbohydrate content in flowers. Moreover, flowers treated with LG at 150 mg L⁻¹ exhibited carbohydrate concentrations of 14.82% and 16.85% in the respective seasons, which, although appreciable, remained lower than the levels achieved with the nanoparticle treatments. In contrast, the control flowers treated with distilled water revealed the lowest carbohydrate content, recording just 11.12% in the first season and 13.37% in the second. These findings highlight the significant influence of Cu-NPs and CHS-NPs in enhancing the carbohydrate content and overall nutritional status of cut flowers.

### Total soluble protein

The results emphasize the total soluble protein content in tulip cut flowers treated with different preservative solutions, including Cu-NPs, CHS-NPs, and LG, are also presented in Fig. 8. The treatment with Cu-NPs at 30 mg L^− 1^ produced the highest total soluble protein levels of 28.87 and 30.45 g kg^− 1^ FW in the first and second seasons, respectively. Displaying it is superior to other treatments. In contrast, the control cut flowers recorded the lowest protein levels at 14.81 and 18.18 g kg^− 1^ FW for the respective seasons. This data indicates that total soluble protein increased across all treatments in the experiment, reinforcing the effectiveness of these preservative solutions compared to the control.

### Antioxidant defense system in cut tulip flowers

#### Superoxide dismutase (SOD) enzymes

**Fig. 9:**
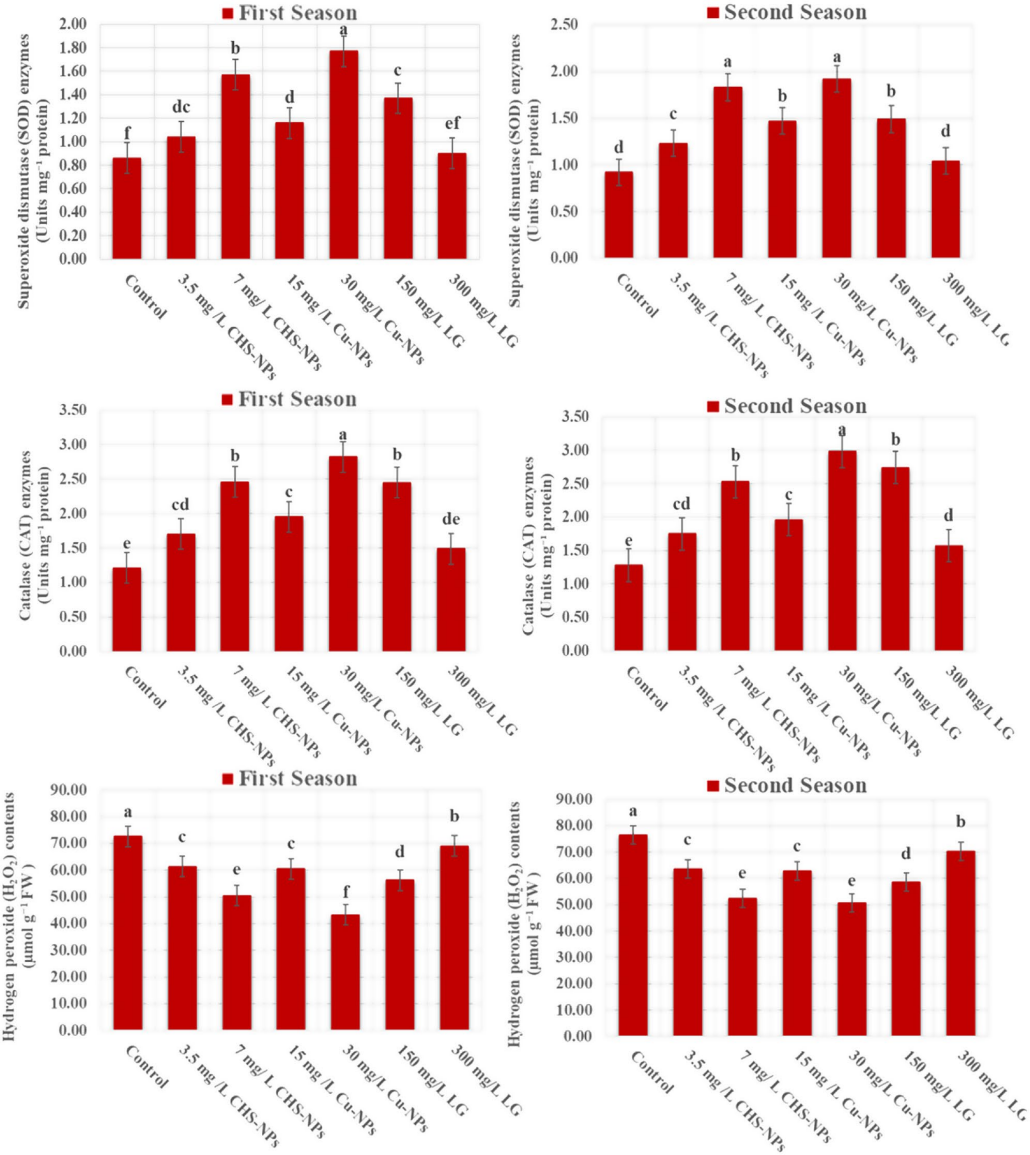
Illustrates the significant impact of varying concentrations of CHS-NPs, Cu-NPs, and LG on the Superoxide dismutase (SOD) and Catalase (CAT) enzymes activity, as well as Hydrogen peroxide (H_2_O_2_) contents in cut tulips over two seasons. Error bars represent the SE of the mean, assessed using Duncan’s multiple range test at *p* ≤ 0.05

As presented in Fig. 9, the application of Cu-NPs at a concentration of 30 mg L⁻¹ significantly (*p* = 0.0001) enhanced superoxide dismutase (SOD) activity in cut tulip flowers compared to the control treatment (distilled water), indicating improved oxidative stress regulation and physiological resilience. Notably, SOD activity exhibited a consistent upward trend throughout the experimental period, with peak values of (1.77 and 1.92 Units mg⁻¹ protein) recorded with the 30 mg L^− 1^ Cu-NPs in the first and second seasons, respectively. In contrast, the control treatment demonstrated substantially lower SOD activity, with values of (0.86 and 0.92 Units mg⁻¹ protein). These results highlight the positive role of Cu-NPs in strengthening the antioxidant defense system and prolonging the vase life of cut tulip flowers.

#### Catalase (CAT) enzymes

Data regarding the effect of NPs, such as Cu and CHS, in addition to LG treatments, on the CAT enzyme activity of cut tulip flowers are presented in Fig. 9. The data indicated highly significant (*p* = 0.0001) differences in CAT enzyme activity in response to different Cu-NPs, CHS-NPs, and LG applications. Notably, the highest number of enzyme units of CAT was recorded when cut tulip flowers were placed in solutions with 30 mg L^− 1^ of Cu-NPs, followed by 7 mg L^− 1^ of CHS- NPs, and then 150 mg L^− 1^ of LG. The numbers of CAT enzyme units (2.82 and 2.98 Units mg^− 1^ protein, in the first and second seasons, respectively) in response to the highest levels of Cu-NPs (30 mg L^− 1^) were significantly higher than those of the controls (1.21 and 1.28 Units mg^− 1^ protein, in the first and second seasons, respectively), indicating a remarkable beneficial effect of Cu-NPs at this concentration. This evidence powerfully supports the usage of Cu-NPs to enhance CAT activity in cut tulips, promoting their longevity and vitality.

#### Hydrogen peroxide (H_2_O_2_) content

The effects of different concentrations of Cu-NPs, CHS-NPs, or LG in floral solutions significantly influenced the hydrogen peroxide (H_2_O_2_) contents in cut tulip flowers, as illustrated in Fig. 9. In both seasons throughout the experiment, H_2_O_2_ levels consistently increased across on-cut tulip flowers in all treatments. Importantly, the control cut flowers in distilled water exhibited the most substantial rise in H_2_O_2_ during both seasons. In contrast, the lowest increase of H_2_O_2_ was recorded in cut flowers supplemented with Cu-NPs at 30 mg L⁻¹ in the first and second seasons. Moreover, the highest recorded H_2_O_2_ content reached 72.64 µmol g^− 1^ FW in the first season and 76.40 µmol g^− 1^ FW in the second season in the control (distilled water) application. This was closely followed by the high concentration of LG at 300 mg L^−^¹, which also displayed remarkable H_2_O_2_ levels in both experimental seasons. These findings demonstrate the crucial role of selecting the appropriate floral solution to significantly improve the longevity and freshness of cut tulip flowers by effectively reducing H_2_O_2_ levels.

#### Heatmap analysis of the antioxidant defense system and total soluble carbohydrate and protein

**Fig. 10:**
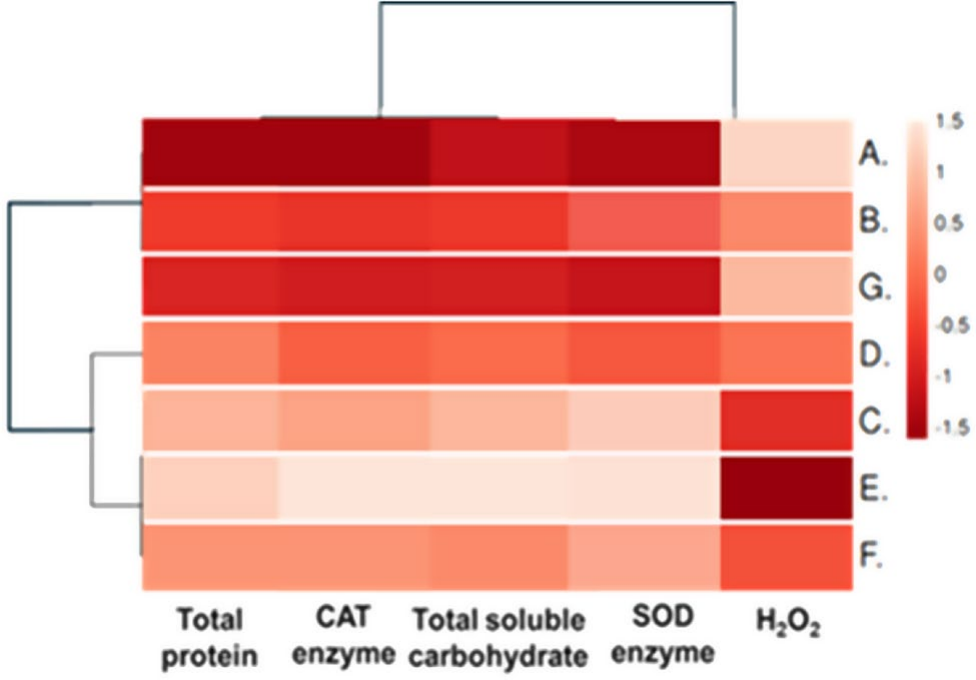
The heatmap Pearson correlation of antioxidant defense system, total soluble carbohydrate, and protein stock traits in cut tulip flowers that were affected by an interaction effect between different concentrations of CHS-NPs, Cu-NPs, or LG in floral solutions, along with the control (distilled water) across experimental conditions

In the heatmap shown in Fig. 10, a strong positive correlation was observed between the traits of the antioxidant defense system and the levels of carbohydrates and proteins in cut tulip flowers under various floral solutions. In contrast, hydrogen peroxide (H₂O₂) levels exhibited negative correlations with carbohydrate and protein levels, as well as with the activities of antioxidant enzymes such as SOD and CAT. This inverse relationship shows that higher oxidative stress markers (H₂O₂) correspond with lower carbohydrate and protein contents and activity of antioxidant enzymes. Therefore, in cut tulip flowers treated with preservation solutions containing Cu-NPs at 30 mg L^-1^, there was a significant increase in total soluble carbohydrates, protein levels, and the activities of CAT and SOD enzymes, and a reduction in H₂O₂ formation. Conversely, in the cut tulip flowers treated with control floral solutions, a decline in antioxidant capacity was observed, marked by decreased CAT and SOD enzyme activity, lower carbohydrate and protein levels, and a significant increase in H₂O₂ levels. These intricate correlations revealed complex interactions among physiological and biochemical processes in response to experimental treatments, providing key insights into the mechanism of stress tolerance and senescence regulation in the studied tulip flower systems.

The first column shows the seven treatments A: Control (distilled water); B: CHS-NPs (3.5 mg L^− 1^), C: CHS-NPs (7 mg L^− 1^), D: Cu- NPs (15 mg L^− 1^), E: Cu-NPs (30 mg L^− 1^), F: LG (150 mg L^− 1^), G: LG (300 mg L^− 1^). The mean data ranged from − 1.5 to 1.5 values of traits [highest (1.5) and lowest (-1.5)]. Also, the color gradient represents the strength and direction of the correlations. Dark Red indicates a low-intensity value of some traits studied; In addition, light red and white indicate a high-intensity value of these traits.

### Mean bacterial counts

**Fig. 11:**
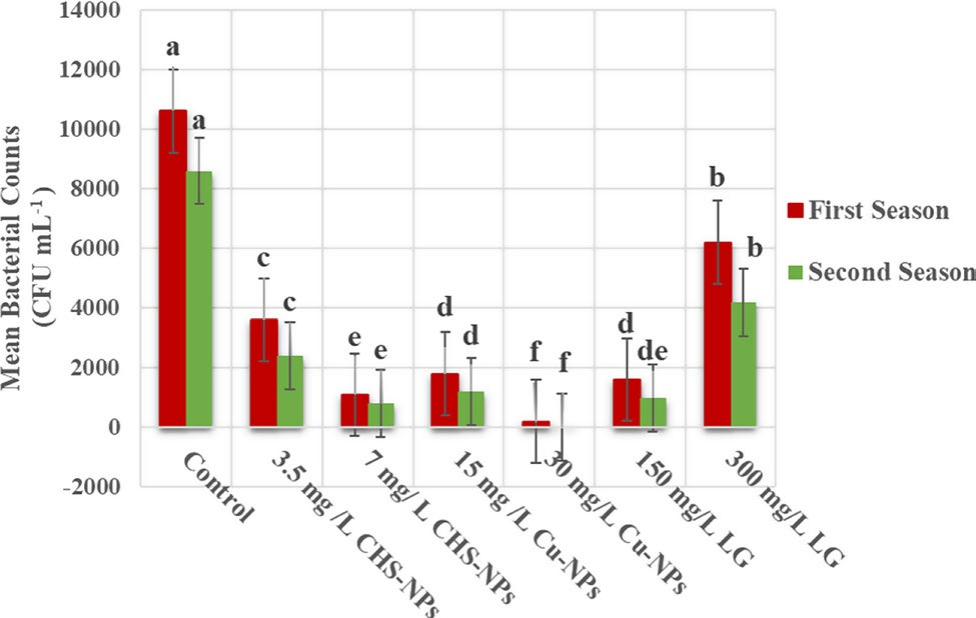
Illustrates the significant impact of varying concentrations of CHS-NPs, Cu-NPs, and LG on bacterial counts (CFU m L^− 1^) in floral solutions used for 7-day postharvest of tulip cut flowers. Error bars represent the SE of the mean, analyzed using Duncan’s multiple range test at a significance level of *p* ≤ 0.05 (ANOVA and Duncan’s multiple range test)

**Fig. 12:**
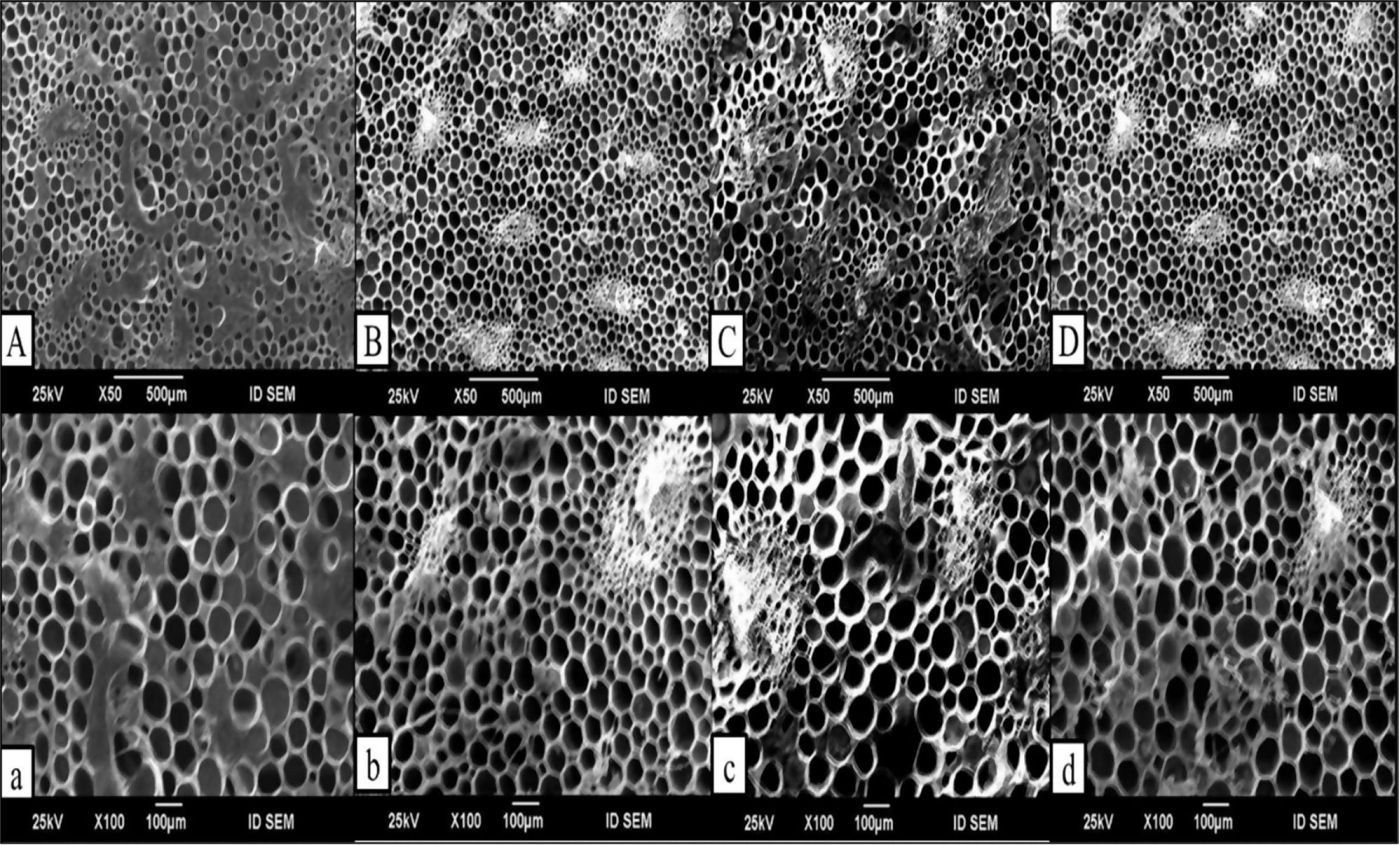
Scanning electron microscope images of tulip xylem vessels in cross-section at the base of the stem compellingly demonstrate how various treatments influence bacterial growth and the resulting blockages in the xylem

Figs. 11 and 12 show the significant (*p* = 0.0000) impact of various treatments on microbial populations in floral solutions during both seasons. Treatments including CHS-NPs, Cu-NPs, and LG solutions effectively reduce bacterial counts compared to the control solution (distilled water). Moreover, in the control solution, mean bacterial counts reached an unacceptable level of 10,600 and 8,600 CFU mL^− 1^ in the first and second seasons, respectively. In contrast, the 30 mg L^−^¹ Cu-NPs treatment decisively reduced these counts to 200 and < 1 CFU mL^− 1^ in the first and second seasons, respectively. Furthermore, the 7 mg L^−^¹ CHS-NPs and 150 mg L^− 1^ LG solutions, also proved effective in significantly lowering bacterial levels throughout both seasons. These results unequivocally demonstrate the superiority of these treatments in combating microbial presence. Additionally, the images in Fig. 12 decisively demonstrate the most significant effectiveness of 30 mg L^−^¹ Cu-NPs in suppressing bacterial growth in floral solutions, followed closely by 7 mg L^−^¹ CHS-NPs and then 150 mg L^−^¹ LG solutions. The 30 mg L^−^¹ Cu-NPs treatment unequivocally outperforms the control and all other tested options in halting microbial proliferation. This compelling evidence strongly demands the acceptance of these treatments to ensure notably more beneficial and vibrant floral solutions.

### Scanning electron microscopy (SEM)

Images from scanning electron microscopy (Fig. 12) indicated that the different concentrations studied of Cu-NPs, CHS-NPs, and LG positively influenced the vase life of cut tulip flowers compared to the control (distilled water). The cross-section of xylem vessel cells in cut flowers treated with distilled water (control) showed significant bacterial accumulation, as illustrated in Fig. 12 (A and a). This bacterial blockage in the xylem leads to water stress, severely limiting the flower’s vase life. As a result of this blockage, the cut flowers were unable to maintain their turgidity, which reduced the vase life of tulip cut flowers. In comparison, flowers treated with CHS-NPs (7 mg L^− 1^), Cu-NPs (30 mg L^− 1^), and LG (150 mg L^− 1^) demonstrated remarkably cleaner xylem vessels and a significant reduction in bacterial accumulation compared to the control. However, it is important to note that the development of bacterial growth and xylem obstructions at the stem end was only slightly diminished by the treatments with Cu-NPs at 30 mg L^− 1^ (Fig. 12, C and c) when evaluated against the control and other treatments. Additionally, using Cu-NPs or CHS-NPs decreases microbial activity, and their ability to effectively inhibit bacterial growth and xylem obstructions at the stem end shows only marginal improvement over the control group and alternative options.

A and a: Control (distilled water), B and b: CHS-NPs (7 mg L^− 1^), C and c: Cu-NPs (30 mg L^− 1^), and D and d: LG (150 mg L^− 1^).

## Discussion

Tulip cut flowers represent a significant source of export revenue within the global floriculture industry. Renowned for their vibrant color diversity, tulips enhance interior spaces and play a vital role in ceremonial occasions such as weddings and funerals. Symbolizing love, appreciation, and respect, they hold considerable cultural and societal importance [56]. It has a relatively short vase life, and its longevity is influenced by numerous factors related to preharvest, harvest, and postharvest practices [76]. Managing these factors is essential for maintaining freshness and quality in the floral industry. Floral preservative solutions are vital in prolonging the longevity of cut flowers, with eco-friendly alternatives offering significant advantages. Recent research has explored various safer, more sustainable options for preserving the postharvest quality and freshness of cut flowers while minimizing environmental and health-related risks [51]. Adopting low-cost, non-toxic substances, such as nanoparticles and essential oils, instead of conventional chemical preservatives represents a promising advancement in floriculture [45, 52]. This approach extends the aesthetic appeal and vitality of flowers and aligns with broader environmental sustainability goals. To achieve the objectives, flower preservative solutions, often formulated with surfactants to maintain optimal water relations and supplemented with potent antioxidant and antimicrobial agents, which are commonly employed to extend the postharvest longevity of cut flowers [28, 50]. 

CHS-NPs, Cu-NPs, and LG oil utilized in this study dramatically enhanced the vase life of tulip cut flowers. By promoting increased water uptake and boosting relative fresh weight (RFW%), these treatments also significantly reduce water loss compared to untreated flowers. The results of this study align with the findings of Rashidiani et al. [60], who proved that Cu-NPs, when applied to a floral solution for cut carnations and chrysanthemums, effectively prevented chlorophyll degradation and extended the vase life. Additionally, cut roses treated with higher levels of Cu-NPs experienced increased vase life due to enhanced antioxidant activity, which helps prevent xylem blockage caused by microbial infections [77]. Furthermore, Cu-NPs improved the relative fresh weight and floral water uptake in cut roses [6]. Similarly, CHS-NPs significantly enhance the vase life of *Rosa hybrid*a plants by effectively reducing transpiration, controlling weight, and delaying ripening while safeguarding the environment from active chemicals [63]. The vase life of cut flowers is crucial for keeping quality. Bañuelos-Hernández et al. [9] found that *Heliconia bihai* lasts up to 20 days with 1.0% CHS treatment, while 1.5% CHS shortens it to 15 days. Spricigo et al. [68] also observed this trend in gerbera-cut flowers, emphasizing the importance of optimal concentration of CHS NPs for longevity. Also, El-Sayed et al. [22] found that CHS-NP treatment enhanced floret opening and increased vase life of cut flowers. Their research also proved the impact of oxidative stress on these flowers, showing that the treatment helped keep photosynthetic pigments and water relations. Consequently, CHS-NPs are an up-and-coming eco-friendly solution for extending the vase life of cut flowers. Furthermore, In *Gerbera jamesonii*, CHS-NPs (110 nm) improved stem water balance and prevented curvature by suppressing microbial growth [68]. Moreover, CuNPs (20 mg L⁻¹) enhanced fresh weight, reduced bacterial populations, and decreased H₂O₂, increasing vase life by 30% in the cut carnation and chrysanthemum flowers. In another study, Dalda Şekerci et al. [16] reported that 30 ppm Cu-NPs applied to cut Narcissus flowers enhanced fresh weight, water uptake, and vase life, like sodium hypochlorite, due to its antimicrobial properties and nourishing effect on the vase solution. Massoud et al. [48] have convincingly shown that treating chrysanthemum cut flowers with a solution containing 25 mg L⁻¹ of LG can significantly prolong their vase life, enhance water uptake, and boost their relative fresh weight. Moreover, Thakur et al. [75] revealed that adding LG to the maintenance of cut gladiolus flowers increases their vase life and improves water absorption. This remarkable improvement is primarily due to citral, an aldehyde in LG, which possesses potent antimicrobial properties that effectively suppress the growth of harmful bacteria [58]. Embracing these findings can elevate floral solutions to new heights.

The current study highlights a crucial connection between key photosynthetic parameters, such as the total chlorophyll index (SPAD), stomatal conductance, and the vase life of cut tulip flowers. These factors are essential for optimizing gas exchange and enhancing the photosynthesis process. Remarkably, our study demonstrates that the application of CHS-NPs, Cu-NPs, and LG significantly boosts both photosynthetic capacity and stomatal conductance, effectively delaying petal senescence and prolonging the vase life of cut tulips. Notably, Cu-NPs at a high concentration of 30 mg L^− 1^ (T5) led to remarkable improvements in stomatal regulation and photosynthetic efficiency by the increase in SPAD value. Recent research by Faraz et al. [25] demonstrates that Cu-NPs enhance plant tissue by increasing total chlorophyll content (SPAD) and boosting the photosynthetic rate, which protects against aging. This improvement is crucial for maintaining chloroplast integrity, preventing oxidative damage, and promoting the biosynthesis of essential photosynthetic pigments, thanks to the protective role of Cu-NPs on chloroplast enzymes [43]. In addition, Cu-NPs effectively inactivate the chlorophyllase enzyme, preventing chlorophyll degradation in chrysanthemums, and extending vase life to an impressive 12 days, compared to just four days for untreated flowers [29]. Similar benefits have been recorded in cut *Rosa hybrida* [63], as well as in cut carnation and chrysanthemum flowers [60]. A similar trend was observed for stomatal conductance, in which Cu-NPs significantly impact photosystem II (PSII), boost the levels of photosynthetic pigments, and improve the ability to assimilate CO_2_, which directly correlates with increased stomatal conductance, photosynthesis rate, and water content in Cut Narcissus flowers [16]. Furthermore, Cu-NPs can attach to or penetrate cell surfaces through pores and lenticels, increasing stomatal conductance, enhancing gas exchange, and further optimizing photosynthesis [55].

In this study, the use of NPs and LG oil led to increases in total soluble carbohydrate and protein of cut tulip leaves compared to the control, which gave the lowest values during both seasons (Fig. 8). Soluble carbohydrates serve as the essential energy source to cut flowers, making them crucial for flower quality because they enhance petal growth, coloration, and longevity while preventing protein degradation; a decline in these carbohydrates can accelerate senescence flowers [13]. Similarly, Soluble proteins are vital for extending the vase life of flowers. Research by Hassan and Ali [30] highlights that higher protein content in cut gladiolus can significantly enhance postharvest longevity because petal senescence is associated with the loss of proteins, which compromises membrane protein integrity and function. Our experiment showed that treating cut tulips with Cu-NPs at 30 mg L^− 1^ (T5), CHS-NPs at 7mg L^− 1^ (T3), or LG at 150 mg L^− 1^(T6) enhances water uptake, Photosynthesis, and sugar translocation, then leads to better resource accumulation and increased turgor pressure, which delays flower senescence. These positive effects have been confirmed in chrysanthemum [29], *Rosa hybrida* [63], and Gladiolus flowers [75], highlighting the potential of these treatments to improve floral longevity.

As shown in Fig. 9 and the heatmap analysis in Fig. 10, these findings suggest that the right nanomaterial can enhance the flowers’ protective enzyme levels, promoting their overall health and longevity. For instance, Sutulienė et al. [72] found that treated plants with Cu-NPs at 12.5 ppm led to notable improvements in the antioxidant system and ROS-scavenging enzyme activity, as well as reduced oxidative stress indicators such as lipid peroxidation and peroxide levels, compared to untreated plants. Similarly, Adhikari et al. [1] reported that Cu-NPs enhanced the production of key antioxidant enzymes, including SOD and POD, resulting in overall higher antioxidant levels. Cu-NPs are essential catalytic centers in plant cellular metabolism. They enhance resistance and defense by boosting antioxidant enzymes like SOD, which converts harmful superoxide anions into H_2_O_2_. Then, CAT transforms H_2_O_2_ into oxygen (O_2_) and water (H_2_O). This process improves chlorophyll content, increases Rubisco activity, and promotes carbohydrate accumulation [78]. In addition, CHS-NPs are distinguished by their unique physical and chemical properties, offering exceptional tensile strength, conductivity, elasticity, and chemical reactivity due to their amine and -OH groups, which enhance water relations [68]. Research by El-Sayed et al. [22, 31] highlights their crucial role in carbohydrate activity for reducing H_2_O_2_ and malondialdehyde levels while mitigating oxidative stress and delaying senescence. Also, as a bioactive material, CHS increases total phenol and sugar contents and boosts antioxidant capacity, helping to counteract oxidative stress following flower harvest [32]. Furthermore, Petriccione et al. [59] found that CHS-NPs enhance enzymatic antioxidants and scavenge H_2_O_2_, improving membrane integrity and significantly enhancing the postharvest quality of loquats.

Mohammadi et al. [51] indicated that Gram-positive bacteria proliferated more rapidly in cut gerbera flowers than Gram-negative bacteria. Over time, both bacterial populations increased in both the vase solutions and the stem tips. This microbial growth significantly compromised the flower’s postharvest quality and vase life, leading primarily to premature wilting and neck bending. in contrast, the present study demonstrates that tulip cut flowers treated with NPs at higher concentrations (T3 and T5) exhibited a significant reduction in bacterial counts compared to the control, which was maintained in distilled water. The findings, clearly illustrated in Figs. 11 and 12, effectively highlight the potential of nanoparticle treatments in enhancing the longevity and overall quality of cut flowers, thereby presenting a valuable advancement in floriculture. Likewise, Rashidiani et al. [60] conducted a study on the use of Cu-NPs at a high concentration of 20 mg L^− 1^ to enhance the longevity of carnation and chrysanthemum cut flowers, the findings indicated significant improvements in RFW%, vase solution uptake, membrane stability index, and total soluble carbohydrates, with a notable reduction in both the bacterial population at the stem end and H_2_O_2_ levels. Esfahani et al. [23] also demonstrated that increasing the concentration of Cu-NPs had a positive impact on the vase life of cut roses, which is the enhancement attributed to the elevated activity of antioxidant enzymes, particularly SOD and CAT, which effectively prevent xylem blockage. Adding Cu-NPs to vases helps flowers last longer by slowly releasing Cu²⁺ ions into the water, and this gradual release keeps harmful bacteria away for a long time during the vase’s life [60]. Cu²⁺ ions are control over enzymes that prevent blockages caused by wounds and slow down the actions of SOD and catalase CAT enzymes [4]. This process reduces the risk of blockages in the xylem at the ends of the flower stems, enhancing the cut flower’s overall longevity and appearance. Additionally, it interacts with biomolecules such as DNA and proteins for the disruption of biochemical processes of bacteria and destruction of the plasma membrane integrity of their cells by the creation of ROS, plus changes in the expression of some apoptosis genes that cause bacterial cell death [71]. As for CHS-NPs, Spricigo et al. [68] provide compelling evidence that a solution containing CHS-NPs was superior in preventing stem bending by effectively controlling microbial growth, significantly inhibiting molds and yeasts on cut gerbera flowers, and outperforming alternative solutions. Furthermore, Seyed Hajizadeh et al. [63] demonstrated that cut roses placed in a preservative solution with CHS-NPs at 5–10 mg L^− 1^ achieved a maximum vase life of 15 days while diminishing microbial growth compared to control solutions. CHs-NPs also benefit from the extended vase life of cut carnations compared to CHS and untreated flowers due to their broad antimicrobial activity against fungal pathogens [66]. However, bulk CHS solubility limitations reduce its effectiveness, while CHS-NPs offer significant advantages by enhancing key properties [34]. Chitosan (CHS) is a safe, biocompatible, renewable, and biodegradable material renowned for its antimicrobial, antifungal, and antioxidant properties. These benefits stem from its degree of deacetylation (DDA), which involves removing acetyl groups from chitin and adding reactive amino groups [19]. This process can enhance cell rupture, disrupt membrane permeability, inhibit bacterial DNA replication, and cause cell death [17]. Thus, the nano-chitosan solution reduced bacterial activity by successfully interacting with the vascular microflora. Moreover, the antimicrobial activity and improved water balance at low concentrations of LG are mainly due to citral, along with minor compounds like limonene, linalool, and myrcene, which act synergistically and may be aided by the oil’s acidity (Thakur et al., 2013). The slight increase in tulip vase life and freshness may stem from LG oil’s slower antimicrobial action and limited water uptake compared to nanoparticle treatments. Nonetheless, Thakur et al. [75] found that low LG concentrations upregulated anti-senescence genes and downregulated pro-senescence genes.

## Conclusion

This study highlights the efficacy of LG, CHS-NPs and Cu-NP as antioxidant and antimicrobial agents in floral preservatives. At concentrations of 30 mg L⁻¹ (Cu-NPs) and 7 mg L⁻¹ (CHS-NPs), these treatments improved water balance, maintained fresh weight, and prevented stem curvature in cut tulips, enhancing overall postharvest quality and longevity. These treatments suppressed microbial growth at the stem base, minimizing xylem blockages and supporting efficient water uptake for up to seven days. Additionally, these treatments significantly increased chlorophyll content, total soluble carbohydrates, and protein levels while reducing hydrogen peroxide (H₂O₂) accumulation and enhancing antioxidant enzyme activity, thereby improving membrane stability. On the other hand, applying LGto the vase solution led to a modest enhancement in both the longevity and visual freshness of cut tulip flowers, which may be linked to improved water balance and antibacterial activity. While LG demonstrated some benefits, its effectiveness was not as pronounced as that of nanoparticle treatments, likely owing to its more gradual antimicrobial action and water uptake. However, the intricate mechanisms through which LG influences flower senescence remain a mystery, inviting further exploration. In summary, the potential of CHS-NPs and Cu-NPs as effective preservative agents in improving post-harvest quality and extending the vase life of cut tulips. Cu-NPs demonstrated superior performance, likely due to their role in enhancing water uptake, activating antioxidant defense pathways, and exerting antimicrobial effects. Using such nanomaterials presents a sustainable, eco-friendly alternative to conventional chemical preservatives, offering a promising advancement for the cut flower industry.

## Data Availability

All data generated or analyzed during this study are included in this published article.

## References

[1] Adhikari T, Sarkar D, Mashayekhi H, Xing B. Growth and enzymatic activity of maize (Zea mays L.) plant: solution culture test for copper dioxide nanoparticles. J Plant Nutr. 2016;39:99–115. 10.1080/01904167.2015.1044012.

[2] Aldhanhani A, Ahmed ZFR. Antioxidant phytochemicals and antibacterial activities of Sidr (*Ziziphus spp*.) leaf extracts. Acta Hortic. 2022;1353:323–32. 10.17660/ActaHortic.2022.1353.40.

[3] Alexieva V, Sergiev I, Mapelli S, Karanov E. The effect of drought and ultraviolet radiation on growth and stress markers in pea and wheat. Plant Cell Environ. 2001;24:1337–44. 10.1046/j.1365-3040.2001.00778.x.

[4] Al-Hakkani MF. Biogenic copper nanoparticles and their applications: A review. SN Appl Sci. 2020;2:505. 10.1007/s42452-020-2279-1.

[5] Ali EF, Issa AA, Al-Yasi HM, Hessini K, Hassan FAS. The efficacies of 1-Methylcyclopropene and Chitosan nanoparticles in preserving the postharvest quality of Damask Rose and their underlying biochemical and physiological mechanisms. Biology. 2022;11:242. 10.3390/biology11020242. 35205108 10.3390/biology11020242 PMC8869683

[6] Amingad V, Sreenivas KN, Fakrudin B, Seetharamu GK, Shankarappa TH, Venugopalan R. Comparison of silver nanoparticles and other metal nanoparticles on postharvest attributes and bacterial load in cut roses var. Taj Mahal Int J Pure App Biosci. 2017;5:579–84. 10.18782/2320-7051.2610.

[7] Armitage AM, Laushman JM. Specialty cut flowers. 2nd ed. OR, USA: Timber Press Portland; 2003.

[8] Asif A, Ali M, Qadir M, Karthikeyan R, Singh Z, Khangura R, Di Gioia F, Ahmed ZF. Enhancing crop resilience by Harnessing the synergistic effects of biostimulants against abiotic stress. Front Plant Sci. 2023;14:1276117. 10.3389/fpls.2023.1276117. 38173926 10.3389/fpls.2023.1276117 PMC10764035

[9] Bañuelos-Hernández KP, García-Nava JR, Leyva-Ovalle OR, Peña-Valdivia CB, Trejo C, Ybarra-Moncada MC. Chitosan coating effect on vase life of flowering stems of *Heliconia bihai* (L.) L. Cv. Halloween. Postharvest Biol Technol. 2017;132:179–87. 10.1016/j.postharvbio.2017.05.009.

[10] Bozzola JJ, Russell LD. Electron microscopy: principles and techniques for biologists. 2nd ed. Boston, USA: Jones and Bartlett Publishers, Inc.; 1999.

[11] Bradford MM. A rapid and sensitive method for the quantitation of microgram quantities of protein utilizing the principle of protein-dye binding. Anal Biochemist. 1976;72:248–54. 10.1016/0003-2697(76)90527-3. 10.1016/0003-2697(76)90527-3 942051

[12] Cakmak I, Marschner H. Magnesium deficiency and high light intensity enhance activities of superoxide dismutase, ascorbate peroxidase, and glutathione reductase in bean leaves. Plant Physiol. 1992;98:1222–7. 10.1104/pp.98.4.1222. 16668779 10.1104/pp.98.4.1222 PMC1080336

[13] Chen C, Chen H, Ni M, Yu F. A study on petal morphological and physiological characteristics of *Styrax japonicus* during the flowering period. J Agron. 2021;11:1498. 10.3390/agronomy11081498.

[14] Colangelo G, Offerhaus A, van Andel T, Stefanaki A. How the wild tulip (Tulipa sylvestris L.) found its way in Northern Europe in the 17th to 19th century: a search through historical gardens and archives. Bot Lett. 2025. 10.1080/23818107.2025.2485447.

[15] Crisan MC, Teodora M, Lucian M. Copper nanoparticles: synthesis and characterization, physiology, toxicity and antimicrobial applications. Appl Sci. 2022;12:141. 10.3390/app12010141.

[16] Dalda Şekerci A, Barut G, Özdal HS, Ünsal HT. The effects of silver and copper nano Particle-Infused vase solutions on the vase life of cut Narcissus (Narcissus L.) flowers. Çukurova J Agric Food Sci. 2024;39:231–40.

[17] Dehnad D, Emam-Djomeh Z, Mirzaei H, Jafari SM, Dadashi S. Optimization of physical and mechanical properties for chitosan–nanocellulose biocomposites. Carbohydr Poly. 2014;105:222–8. 10.1016/j.carbpol.2014.01.094. 10.1016/j.carbpol.2014.01.094 24708973

[18] Duncan DB. Multiple ranges and multiple F. test. Biometrics. 1955;11:1–42. 10.2307/3001478.

[19] El Ghaouth A, Arul J, Wilson C, Benhamou N. Biochemical and cytochemical aspects of the interactions of Chitosan and Botrytis cinerea in bell pepper fruit. Postharvest Biol Technol. 1997;12:183–94. 10.1016/S0925-5214(97)00056-2.

[20] Elhindi KM. Evaluation of several holding solutions for prolonging vase-life and keeping quality of cut sweet pea flowers (*Lathyrus odoratus* L). Saudi J Biol Sci. 2012;19:195–202. 10.1016/j.sjbs.2011.12.001. 23961179 10.1016/j.sjbs.2011.12.001 PMC3730932

[21] El-Sayed IM, El-Ziat RA. Utilization of environmentally friendly essential oils on enhancing the postharvest characteristics of Chrysanthemum morifolium Ramat cut flowers. Heliyon. 2021;7:e05909. 10.1016/j.heliyon.2021.e05909. 33521350 10.1016/j.heliyon.2021.e05909 PMC7820481

[22] El-Sayed IM, Salim RG, El-Haggar EF, El-Ziat RA, Soliman DM. Molecular characterization and positive impact of brassinosteroids and Chitosan on *Solidago canadensis* Cv. Tara Characteristics Horticulturae. 2020;6:100. 10.3390/horticulturae6040100.

[23] Esfahani MB, Pour MJ, Mortazaeinezhad F, Saeed SE. The effect of nano copper, nanosilver and sucrose on vase life of cut Rose dolcevita’. Inter J Agri Crop Sci. 2013;5:36–8.

[24] Etheredge CL, Waliczek TM, DelPrince J. Comparison of united States consumers’ perceptions and willingness to pay for sustainable environmental practices in the retail floral industry based on geographical regions. HortTechnol. 2024;34:241–51. 10.21273/HORTTECH05392-24.

[25] Faraz A, Faizan M, Rajput VD, Minkina T, Hayat S, Faisal M, Alatar AA, Abdel-Salam EM. CuO Nanoparticle-Mediated seed priming improves Physio-Biochemical and enzymatic activities of *Brassica juncea*. Plants. 2023;12:803. 10.3390/plants12040803. 36840152 10.3390/plants12040803 PMC9959013

[26] Francis DV, Abdalla AK, Mahakham W, Sarmah AK, Ahmed ZFR. Interaction of plants and metal nanoparticles: exploring its molecular mechanisms for sustainable agriculture and crop improvement. Environ Int. 2024;190:108859. 10.1016/j.envint.2024.108859 10.1016/j.envint.2024.108859 38970982

[27] Giannopolitis CN, Ries SK. Superoxide dismutase occurrence in higher plants. Plant Physiol. 1977;59:309–14. 10.1104/pp.59.2.309. 16659839 10.1104/pp.59.2.309 PMC542387

[28] Gururani MA, Atteya AK, Elhakem A, El-Sheshtawy ANA, El-Serafy RS. Essential oils prolonged the cut carnation longevity by limiting the xylem blockage and enhancing the physiological and biochemical levels. PLoS ONE. 2023;3:e0281717. 10.1371/journal.pone.0281717. 10.1371/journal.pone.0281717 PMC9990951 36881583

[29] Hashemabadi D, Kaviani B, Shirinpour A, Zahiri S. Effects of copper nanoparticles (CNPs) on vase life of cut flowers chrysanthemum (*Chrysanthemum morifolium* L.‘White’). Eur J Exp Biol. 2013;3:153–5.

[30] Hassan FAS, Ali EF. Protective effects of 1-methylcyclopropene and Salicylic acid on senescence regulation of gladiolus cut spikes. Sci Hort. 2014;179:146–52. 10.1016/j.scienta.2014.09.025.

[31] Hassan FAS, Ali EF, Mostafa NY, Mazrou R. Shelf-life extension of sweet Basil leaves by edible coating with thyme volatile oil encapsulated Chitosan nanoparticles. Int J Biol Macromol. 2021;177:517–25. 10.1016/j.ijbiomac.2021.02.159. 33636264 10.1016/j.ijbiomac.2021.02.159

[32] Hong-Juan J, Huan-Qing L. Chitooligosaccharide prolongs vase life of cut roses by decreasing reactive oxygen species. Korean J Hortic Sci Technol. 2015;33:383–9. 10.7235/hort.2015.14188.

[33] Irigoyen JJ, Einerich DW, Sánchez-Díaz M. Water stress induced changes in concentrations of proline and total soluble sugars in nodulated alfalfa (*Medicago sativa*) plants. Physiol Plant. 1992;84:55–60. 10.1111/j.1399-3054.1992.tb08764.x.

[34] Iriti M, Varoni EM. Chitosan-induced antiviral activity and innate immunity in plants. Environ Sci Pollut Res. 2015;22:2935–44. 10.1007/s11356-014-3571-7. 10.1007/s11356-014-3571-7 25226839

[35] Iwaya-Inoue M, Tataka M. Trehalose plus Chloramphenicol prolong the vase life of tulip flowers. HortSci. 2001;36:946–50. 10.21273/HORTSCI.36.5.946.

[36] Jahnke NJ, Kalinowski J, Dole JM. Postharvest handling techniques for Long-term storage of cut tulip and Dutch Iris. HortTechnol. 2022;32:263–74. 10.21273/HORTTECH05010-21.

[37] Joyce DC, Jones PN. Water balance of the foliage of cut Geraldton waxflower. Postharvest Biol Technol. 1992;2:31–9. 10.1016/0925-5214(92)90025-K.

[38] Kaur N, Shahwar D, Hassan FE, Ahmed ZFR. Antioxidant and antibacterial activities of date palm fruit (*Phoenix dactylifera* L.) in response to postharvest application with natural elicitors. Acta Hortic. 2023;1364:187–94. 10.17660/ActaHortic.2023.1364.25.

[39] Kaur N, Somasundram C, Razali Z, Mourad A-HI, Hamed F, Ahmed ZFR. *Aloe vera*/Chitosan-Based edible film with enhanced antioxidant, antimicrobial, thermal, and barrier properties for sustainable food preservation. Polymers. 2024;14:242. 10.3390/polym16020242. 10.3390/polym16020242 PMC10821446 38257041

[40] Khairy AM, Tohamy MR, Zayed MA, Mahmoud SF, El-Tahan AM, El-Saadony MT, Mesiha PK. Eco-friendly application of nano-chitosan for controlling potato and tomato bacterial wilt. Saudi J Biol Sci. 2022;29:2199–209. 10.1016/j.sjbs.2021.11.041. 35531227 10.1016/j.sjbs.2021.11.041 PMC9073058

[41] Khan W, Prithviraj B, Smith DL. Photosynthetic responses of corn and soybean to foliar application of salicylates. J Plant Physiol. 2003;160:485–92. 10.1078/0176-1617-00865. 12806776 10.1078/0176-1617-00865

[42] Khatri D, Panigrahi J, Prajapati A, Bariya H. Attributes of Aloe vera gel and Chitosan treatments on the quality and biochemical traits of post-harvest tomatoes. Sci Hort. 2020;259:108837. 10.1016/j.scienta.2019.108837.

[43] Liu A, Xiao W, Lai W, Wang J, Li X, Yu H, Zha Y. Potential application of selenium and copper nanoparticles in improving growth, quality, and physiological characteristics of strawberry under drought stress. Agriculture. 2024;14:1172. 10.3390/agriculture14071172.

[44] Lü P, Huang X, Li H, Liu J, He S, Joyce DC, Zhang Z. Continuous automatic measurement of water uptake and water loss of cut flower stems. Hortsci. 2011;46:509–12. 10.21273/HORTSCI.46.3.509.

[45] Manzoor A, Bashir MA, Hashmi MM. Nanoparticles as a preservative solution can enhance postharvest attributes of cut flowers. Italus Hortus. 2020;27:1–14. 10.26353/j.itahort/2020.2.0114.

[46] Marasek-Ciolakowska A, Sochacki D, Marciniak P. Breeding aspects of selected ornamental bulbous crops. Agronomy. 2021;11:1709. 10.3390/agronomy11091709.

[47] Marousky FJ. Conditioning gladiolus spikes to maintenance of fresh weight with pre-treatments of 8-hydroxy-quinoline citrate plus sucrose. Proc Fla State Hortic Soc. 1970;82:411–4.

[48] Massoud HYA, Kassem MM, Farag NBB. Effect of some essential oils on cut flowers of chrysanthemum (*Dendranthema grandiflorum* Ram.) cv.flyer. J Plant Prod. 2015;6:563–74. 10.21608/jpp.2015.49581.

[49] Metsalu T, Vilo J. ClustVis: A web tool for visualizing clustering of multivariate data using principal component analysis and heatmap. Nucleic Acids Res. 2015;43:W566–70. 10.1093/nar/gkv468. 25969447 10.1093/nar/gkv468 PMC4489295

[50] Mohammadi M, Aelaei M, Saidi M. Pre-harvest and pulse treatments of spermine, γ- and β-aminobutyric acid increased antioxidant activities and extended the vase life of gerbera cut flowers ‘stanza’. Ornam Hortic. 2020a;26(2):305–16. 10.1590/2447-536X.v26i2.2120.

[51] Mohammadi M, Aelaei M, Saidi M. Antibacterial properties of *Scrophularia striata* boiss. (Tashenehdari) extract on vase life improvement in stanza and Pink elegance gerbera cut flowers. Biocataly Agricultural Biotechnol. 2020;28:101738. 10.1016/j.bcab.2020.101738.

[52] Mohammadi M, Eghlima G, Ranjbar M-E. Ascorbic acid reduces chilling injury in anthurium cut flowers during cold storage by increasing Salicylic acid biosynthesis. Postharvest Biology Technol. 2023;201:112359. 10.1016/j.postharvbio.2023.112359.

[53] Moussa MM, Mazrou RM, Hassan FAS. How Sage and Rosemary essential oils regulate postharvest senescence and extend the vase life of cut Gladiolus spikes. Horticulturae. 2024;10:638. 10.3390/horticulturae10060638.

[54] Mutlu-Ingok A, Devecioglu D, Dikmetas DN, Karbancioglu-Guler F, Capanoglu E. Antibacterial, antifungal, antimycotoxigenic, and antioxidant activities of essential oils: an updated review. Molecules. 2020;25:4711. 10.3390/molecules25204711. 33066611 10.3390/molecules25204711 PMC7587387

[55] Nekoukhou M, Fallah S, Pokhrel LR, Abbasi-Surki A, Rostamnejadi A. Foliar enrichment of copper oxide nanoparticles promotes biomass, photosynthetic pigments, and commercially valuable secondary metabolites and essential oils in Dragonhead (*Dracocephalum Moldavica* L.) under semi-arid conditions. Sci Total Environ. 2023;863:160920. 10.1016/j.scitotenv.2022.160920. 36529390 10.1016/j.scitotenv.2022.160920

[56] Nguyen TK, Lim JH. Do Eco-Friendly floral preservative solutions prolong vase life better than chemical solutions? Horticulturae. 2021;7:415. 10.3390/horticulturae7100415.

[57] Othman EZ, Esmail SEA. Enhancing vase life of *Helianthus annuus* L. cut flowers using Salicylic acid and dill essential oil. Middle East J Agri Res. 2020;9:1045–56. 10.36632/mejar/2020.9.4.81.

[58] Peichel C, Nair DVT, Dewi G, Donoghue AM, Reed KM, Kollanoor JA. Effect of Lemongrass (*Cymbopogon citratus*) essential oil on the survival of Multidrug-Resistant Salmonella enterica serovar Heidelberg in contaminated poultry drinking water. J Appl Poult Res. 2019;28:1121–30. 10.3382/japr/pfz076.

[59] Petriccione M, Pagano L, Forniti R, Zampella L, Mastrobuoni F, Scortichini M, Mencarelli F. Postharvest treatment with Chitosan affects the antioxidant metabolism and quality of wine grape during partial dehydration. Postharvest Biol Technol. 2018;137:38–45. 10.1016/j.postharvbio.2017.11.010.

[60] Rashidiani N, Nazari F, Javadi T, Samadi S. Copper nanoparticles (CuNPs) increase the vase life of cut carnation and Chrysanthemum flowers: antimicrobial ability and morphophysiological improvements. Ornam Hortic. 2020;26:225–35. 10.1590/2447-536X.v26i2.2156.

[61] Schweitzer B, Balázs VL, Molnár S, Szögi-Tatár B, Böszörményi A, Palkovics T, Horváth G, Schneider G. Antibacterial effect of Lemongrass (*Cymbopogon citratus*) against the aetiological agents of pitted keratolyis. Molecules. 2022;27:1423. 10.3390/molecules27041423. 35209211 10.3390/molecules27041423 PMC8878996

[62] Solgi M. Evaluation of plant-mediated silver nanoparticles synthesis and its application in postharvest Physiology of cut Flowers. Physiol Mol Biol Plants. 2014;20:279–285. 10.1007/s12298-014-0237-3 25049454 10.1007/s12298-014-0237-3 PMC4101138

[63] Seyed Hajizadeh H, Dadashzadeh R, Azizi S, Reza Mahdavinia G, Kaya O. Effect of Chitosan nanoparticles on quality indices, metabolites, and vase life of *Rosa hybrida* cv. Black magic. Chem Biol Technol Agric. 2023;10:12. 10.1186/s40538-023-00387-7.

[64] Sharma P, Bhargava B, Sangmesh P, Ujala. Agro-Biodiversity: conservation and use of plant genetic resources. In: Datta SK, Gupta YC, editors. Floriculture and ornamental plants. Handbooks of crop diversity: conservation and use of plant genetic resources. Singapore: Springer; 2022. 10.1007/978-981-15-3518-5_9.

[65] Skutnik E, Rabiza-Świder J, Jędrzejuk A, Łukaszewska A. The effect of the Long-Term cold storage and preservatives on senescence of cut herbaceous peony flowers. Agronomy. 2020;10:1631. 10.3390/agronomy10111631.

[66] Solgi M. The application of new environmentally friendly compounds on postharvest characteristics of cut carnation (*Dianthus caryophyllus* L). Rev Bras Bot. 2018;41:515–22. 10.1007/s40415-018-0464-x.

[67] Song J, Li Y, Hu J, Lee J, Jeong BR. Pre- and/or postharvest silicon application prolongs the vase life and enhances the quality of cut peony (*Paeonia lactiflora* Pall.) flowers. Plants. 2021;10:1742. 10.3390/plants10081742. 34451787 10.3390/plants10081742 PMC8398881

[68] Spricigo PC, Pilon L, Trento JP, de Moura MR, Bonfim KS, Mitsuyuki MC, Mattoso LHC, Ferreira MD. Nano-chitosan as an antimicrobial agent in preservative solutions for cut flowers. J Chem Technol Biotechnol (JCTB). 2021;96:2168–75. 10.1002/jctb.6766.

[69] Spricigo PC, Trento JP, Bresolin JD, Soares VF, Ferraz LDM, Ferreira MD. Methods of Preparing flower stem samples for scanning electron microscopy. Artigos Técnicos. 2015;21:17–26. 10.14295/rbho.v21i1.771.

[70] Stefanaki A, Walter T, van Andel T. Tracing the introduction history of the tulip that went wild (*Tulipa sylvestris*) in sixteenth-century Europe. Sci Rep. 2022;12:9786. 10.1038/s41598-022-13378-9. 35697708 10.1038/s41598-022-13378-9 PMC9192774

[71] Sun T, Yan Y, Zhao Y, Guo F, Jiang C. Copper oxide nanoparticles induce autophagic cell death in A549 cells. PLoS ONE. 2012;7:e43442. 10.1371/journal.pone.0043442. 22916263 10.1371/journal.pone.0043442 PMC3423358

[72] Sutulienė R, Ragelienė L, Duchovskis P, Miliauskienė J. The effects of Nano-copper, -molybdenum, -boron, and -silica on pea (Pisum sativum L.) growth, antioxidant properties, and mineral uptake. J Soil Sci Plant Nutr. 2022;22:801–14. 10.1007/s42729-021-00692-w.

[73] Tayal R, Kumar V, Irfan M. Harnessing the power of hydrogen sulphide (H_2_S) for improving fruit quality traits. Plant Biol. 2021;24:594–601. 10.1111/plb.13372. 34866296 10.1111/plb.13372

[74] Teerarak M, Pilasombut K, Laosinwattana C. Peppermint essential oil enhances the vase life of Dendrobium orchids. Heliyon. 2024;10:e31636. 10.1016/j.heliyon.2024.e31636. 38845939 10.1016/j.heliyon.2024.e31636 PMC11153112

[75] Thakur M, Verma V, Chandel A, Kumar R, Sharma T, Kumar A, Bhardwaj S, Kumar R, Bhargava B. Lemon grass essential oil improves *Gladiolus grandiflorus* postharvest life by modulating water relations, microbial growth, biochemical activity, and gene expression. Sci Rep. 2023;13:2630. 10.1038/s41598-023-28829-0. 36788264 10.1038/s41598-023-28829-0 PMC9929329

[76] Ullah MJ, Bashir M, Gul H, Shahzad A, Shahzad M. Use of citric acid and Iron sulfate in promoting Post-Harvest longevity of cut tulips (Tulipa gesneriana L. Cv. Marylin) in vase solutions. Contemp Agri. 2022;71:57–64. 10.2478/contagri-2022-0009.

[77] Vahidi P, Jafarpour M. Effects of herbal essences and Nano-technology on enzyme activity of cut Rose flowers cultivar ‘black majic’. Specialty J Biol Sci. 2015;1:14–8.

[78] Wu H, Shabala L, Shabala S, Giraldo JP. Hydroxyl radical scavenging by cerium oxide nanoparticles improves Arabidopsis salinity tolerance by enhancing leaf mesophyll potassium retention. Environ Sci Nano. 2018;5:1567–83. 10.1039/C8EN00323H.

[79] Xylia P, Chrysargyris A, Shahwar D, Ahmed ZFR, Tzortzakis N. Application of Rosemary and Eucalyptus essential oils on the preservation of cucumber fruit. Horticulturae. 2022;8:774. 10.3390/horticulturae8090774.

[80] Yaquby AM, Ahmadi BA, Ashkar SB. Effects of plant growth regulators on quality, quantity and Vase-life of Rose flower (*Rosa hybrida* cv. Avalanche). J Humanit Social Sci Stud. 2022;4:206–12. 10.32996/jhsss.2022.4.3.20.

[81] Zafar A, Ahmad I, Ahmad A, Ahmad M. Copper (II) oxide nanoparticles augment antifilarial activity of albendazole: in vitro synergistic apoptotic impact against filarial parasite *Setaria cervi*. Int J Pharma. 2016;50:49–64. 10.1016/j.ijpharm.2016.01.059. 10.1016/j.ijpharm.2016.01.059 26827921

[82] Zahedi SM, Karimi M, Da Silva JAT. The use of nanotechnology to increase quality and yield of fruit crops. J Sci Food Agri. 2020;100:25–31. 10.1002/jsfa.10004. 10.1002/jsfa.10004 31471903

